# Effective delivery of miR-150-5p with nucleus pulposus cell-specific nanoparticles attenuates intervertebral disc degeneration

**DOI:** 10.1186/s12951-024-02561-x

**Published:** 2024-05-27

**Authors:** Hua Jiang, Hongyu Qin, Qinghua Yang, Longao Huang, Xiao Liang, Congyang Wang, Abu Moro, Sheng Xu, Qingjun Wei

**Affiliations:** 1 Department of Spine Surgery, The First Affiliated Hospital of Guangxi Medical University, 6 Shuangyong Road, Nanning, 530021 Guangxi Zhuang Autonomous Region People’s Republic of China; 2 Department of Orthopaedic Surgery, The First Affiliated Hospital of Guangxi Medical University, 6 Shuangyong Road, Nanning, 530021 Guangxi Zhuang Autonomous Region People’s Republic of China; 3 Research Centre for Regenerative Medicine, Guangxi Engineering Center in Biomedical Material for Tissue and Organ Regeneration, Guangxi Medical University, 22 Shuangyong Road, Nanning, 530021 Guangxi Zhuang Autonomous Region People’s Republic of China

**Keywords:** Intervertebral disc degeneration, microRNA, Nanoparticles, Ubiquitination

## Abstract

**Background:**

The use of gene therapy to deliver microRNAs (miRNAs) has gradually translated to preclinical application for the treatment of intervertebral disc degeneration (IDD). However, the effects of miRNAs are hindered by the short half-life time and the poor cellular uptake, owing to the lack of efficient delivery systems. Here, we investigated nucleus pulposus cell (NPC) specific aptamer-decorated polymeric nanoparticles that can load miR-150-5p for IDD treatment.

**Methods:**

The role of miR-150-5p during disc development and degeneration was examined by miR-150-5p knockout (KO) mice. Histological analysis was undertaken in disc specimens. The functional mechanism of miR-150-5p in IDD development was investigated by qRT-PCR assay, Western blot, coimmunoprecipitation and immunofluorescence. NPC specific aptamer-decorated nanoparticles was designed, and its penetration, stability and safety were evaluated. IDD progression was assessed by radiological analysis including X-ray and MRI, after the annulus fibrosus needle puncture surgery with miR-150-5p manipulation by intradiscal injection of nanoparticles. The investigations into the interaction between aptamer and receptor were conducted using mass spectrometry, molecular docking and molecular dynamics simulations.

**Results:**

We investigated NPC-specific aptamer-decorated polymeric nanoparticles that can bind to miR-150-5p for IDD treatment. Furthermore, we detected that nanoparticle-loaded miR-150-5p inhibitors alleviated NPC senescence in vitro, and the effects of the nanoparticles were sustained for more than 3 months in vivo. The microenvironment of NPCs improves the endo/lysosomal escape of miRNAs, greatly inhibiting the secretion of senescence-associated factors and the subsequent degeneration of NPCs. Importantly, nanoparticles delivering miR-150-5p inhibitors attenuated needle puncture-induced IDD in mouse models by targeting FBXW11 and inhibiting TAK1 ubiquitination, resulting in the downregulation of NF-kB signaling pathway activity.

**Conclusions:**

NPC-targeting nanoparticles delivering miR-150-5p show favorable therapeutic efficacy and safety and may constitute a promising treatment for IDD.

**Graphical Abstract:**

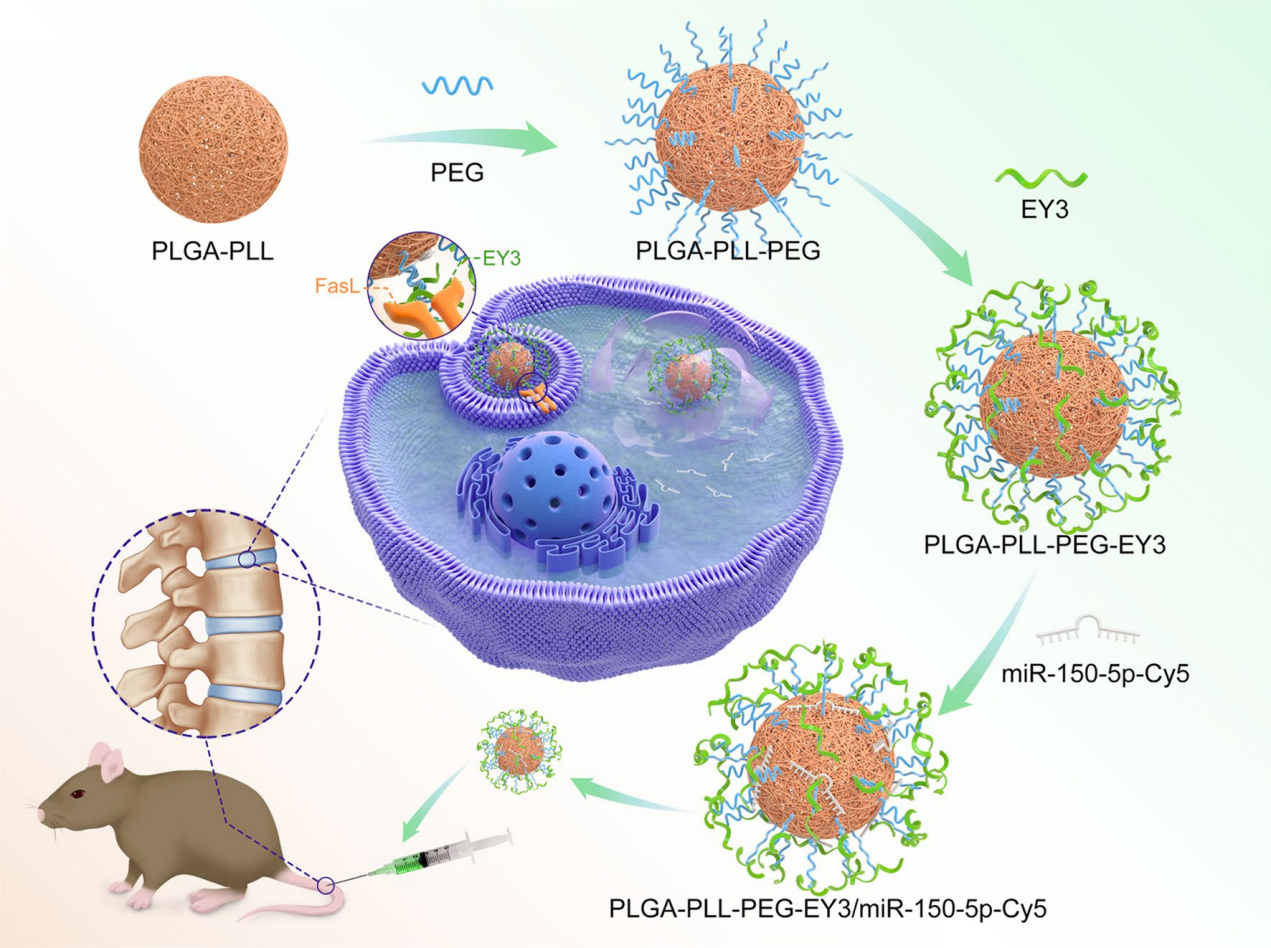

**Supplementary Information:**

The online version contains supplementary material available at 10.1186/s12951-024-02561-x.

## Background

Intervertebral disc degeneration (IDD) is one of the most common degenerative musculoskeletal disorders in the world [1] and is characterized by accelerated senescence, decreased proliferation, and enhanced apoptosis of nucleus pulposus cells (NPCs) [2, 3]. These factors may lead to disc dehydration, disc space narrowing and disc bulging, resulting in low back pain and leg disability [4]. Current treatment options for IDD, such as medication, physical therapy, and invasive surgery [5], mainly focus on symptom alleviation and cannot reverse the underlying pathological changes. Since both surgical and nonsurgical treatment options produce suboptimal outcomes in most cases, there is therefore a need to explore other effective therapeutic strategies for IDD therapy. To address this drawback, gene therapy is considered an optimal therapeutic strategy for restoring the physiological and biomechanical structure of intervertebral discs [6].

MicroRNAs (miRNAs) are a type of single-stranded noncoding RNA with a length of 18–24 nucleotides that can regulate the activity of up to 50% of coding genes in mammals [7]. Emerging evidence suggests that miRNAs play a vital role in the pathophysiological processes of IDD through complex intracellular signaling networks [8, 9]. This property renders miRNAs highly interesting therapeutic targets for restoring NPC functions and reversing IDD phenotypes. Recently, miRNA-based therapeutics have gradually translated from basic research to clinical application in the field of degenerative musculoskeletal diseases [10–15], which offers novel insights into precise miRNA-targeted treatments for IDD. However, the lack of an efficient NPC-specific delivery system hinders the application of miRNA-based therapeutics in vivo [16, 17].

With the rapid developments of nanomedicine, many biological delivery systems have been investigated for transferring miRNAs to treat IDD, including organic (polymeric or lipidic) nanoparticles [18–22], inorganic nanoparticles [23, 24], nano-hydrogels [25–27], and extracellular vesicles (EVs) [28–30]. Different delivery systems possess different biological and release characteristics, and therefore hold promise for advancing the treatment of IDD [17, 31]. For example, in vitro and in vivo studies have evaluated EVs characterization metrics, delivery methods, safety and efficacy profiles [32, 33]. It has been suggested that EVs-based therapeutics targeting IDD offer the advantages of biocompatibility and immunogenicity, as well as a more straightforward pathway to regulatory approval [34]. In addition to EVs, polymeric nanoparticles have emerged as an attractive nanodrug vehicle, offering superior control over their structure, mechanical properties, and composition. Polymeric nanoparticles are easier to chemically modify, can be customized for specific applications, and can be manufactured on a large scale to provide renewable possibilities [18]. In our preclinical animal models of osteoarthritis [12, 13], polymeric nanoparticles combined with aptamer conjugates could enhance intracellular uptake, reduce cellular apoptosis and increase extracellular matrix (ECM) production. It provides a new cell-targeted drug delivery option for precise IDD treatment.

In this work, we equipped optimally charged poly-l-lysine (PLL)-poly (l-lactide-co-glycolide) (PLGA)-polyethylene glycol (PEG) dendrimers with an NPC-specific aptamer that could directly bind to the transmembrane protein FasL on the surface of NPCs, thereby accelerating the transport of nanoparticles to NPCs. To the best of our knowledge, this study may be the first to report the use of NPC-specific aptamer (EY3)-PLGA-PLL-PEG nanoparticles encapsulating miR-150-5p as a therapeutic option for IDD. Our findings provide evidence and could lead to new therapeutic perspectives on the pharmacological inhibition of miR-150-5p as a disc degradation amelioration option, thus offering a promising nanotechnology-based precision-targeting treatment for IDD.

## Methods

### Human subjects

According to the clinical disc tissue bank, a total of 30 control subjects who underwent spinal decompression and internal fixation via anterolateral approaches were selected for the control group. Moreover, age- and sex-matched nucleus pulposus (NP) tissues were collected from 67 confirmed IDD patients who underwent lumbar interbody fusion surgery. The Pfirrmann grade (using preoperative MRI scans) and histological examination of the NP specimens were subsequently performed for both the IDD and control groups. All IDD patients were classified as having severe degeneration (grades III–IV), whereas all controls were classified as having mild degeneration (grades I–II). (Supplementary Table 1) This study was approved by the Ethics Committee of the First Affiliated Hospital of Guangxi Medical University (No. 2023-S278-01), and informed consent was obtained from all participants included in the study.

### Knockout mouse models

Heterozygous knockouts (KOs) of miR-150-5p in the C57BL/6 background were obtained from Jackson Laboratories (Bar Harbor, ME, USA). Global miR-150-5p knockout (miR-150-5p KO) mice were purchased from GemPharmatech Co., Ltd. (Nanjing, China; production license number: GJS021901015). Conventional mouse tail DNA genotyping was subsequently performed to confirm the specific deletion of miR-150-5p in the NP tissue. All mice used in this study were kept in hygienic pathogen-free conditions, with a maximum of 5 mice per cage. The experimental protocol used in this study was approved by the Animal Experimentation Care and Use Committee of the First Affiliated Hospital of Guangxi Medical University (No. 2023-S278-01), and all the experiments were in accordance with the regulations of the committee.

### miRNA microarray analysis

A human miRNA microarray slide (miRBase Database V21.0) containing 2,549 human miRNAs (8 × 60 k slide formats printed using Agilent′s 60-mer SurePrint technology) was used for miRNA expression profiling. Sample labeling and array hybridization were performed in accordance with the Agilent miRNA Microarray System and miRNA Complete Labeling and Hyb Kit protocol (Agilent Technologies, Santa Clara, CA, USA). Using Cyanine 3-pCp in combination with T4 RNA ligase, the total miRNA in each sample was labeled. The labeled cRNAs then underwent inspissation and desiccation before being redissolved in water. One microgram of labeled cRNAs was fragmented using 11 μl of 10 × blocking agent and 2.2 μl of fragmentation buffer (25 ×), heated at 60 °C for 30 min and finally diluted with 55 μl of hybridization buffer (2 ×). Then, 100 μl of hybridization solution was distributed onto gasket slides, which were subsequently assembled onto gene expression microarray slides. The slides were then incubated at 65 °C for 17 h. in an Agilent hybridization oven. Agilent Feature Extraction software (version 11.0.1.1) was subsequently used to analyze the acquired array images. The GeneSpring GX (v12.1) software package (Agilent Technologies, Santa Clara, CA, USA) was used for data standardization and subsequent data processing. Following data standardization, flagged miRNAs (those with a “All Targets Value”) identified in at least 3 of the 6 samples were selected for further analysis. The miRNAs with statistically significant differential expression between the two groups were subsequently screened using a volcano plot. Using fold change screening, the differentially expressed miRNAs were identified in the two samples, and hierarchical clustering was performed using R scripts.

#### In vitro experiments

##### Cell culture and transfection

Primacy NPCs were isolated from human intervertebral disc (IDD patients and controls), mouse intervertebral disc (miR-150-5p KO and C57BL/6 wild-type mice). NPCs were preserved as a monolayer in high-glucose Dulbecco’s modified Eagle’s medium (DMEM) containing 10% fetal bovine serum (FCS), 100 IU/ml penicillin, and 100 μg/ml streptomycin in an atmosphere of 5% carbon dioxide at 37 °C. All the experiments involved the use of human NPCs at a confluence rate of 85%. As previously described [10], the cells were inoculated into 96-well plates for three-dimensional culture of the NPCs. The NPCs were then cultured at 37 °C and 5% carbon dioxide in 200 µl of NPC growth medium. After 21 days of culture, the cells were collected for further evaluation. Using a Silencer® siRNA labeling kit or miRNA negative control (mirVana miRNA mimics/inhibitor negative control #1) (Life Technologies), Lipofectamine RNAiMAX transfection reagent (Invitrogen, Carlsbad, CA, USA), and Cy3-labeled or unlabeled miR-150-5p (a mirVana miRNA mimic or inhibitor) were transfected into human NPCs, SW1353 cells, or C28/I2 cells at 50 nM. The FBXW11 expression plasmid (pcDNA 3.1/V5-His TOPO TA Expression Kit) (Invitrogen) was subsequently obtained.

##### miR-150-5p knockout (KO) NPCs and gene expression microarray

In vitro miR-150-5p KO human NPCs were generated using the CRISPR/Cas9 system (Santa Cruz Biotechnology, Inc.). In accordance with the manufacturer′s instructions, CRISPR/Cas9 KO plasmid and control CRISPR/Cas9 plasmid transfections were performed for miR-150-5p. The Whole-Human Genome Oligo Microarray covering 27,958 Entrez gene RNAs was used to screen for differentially expressed genes. The Agilent monochrome microarray gene expression analysis program (Agilent Technologies, Santa Clara, CA, USA) was used to perform the sample labeling and chip hybridization. In brief, the total RNA of each sample was linearly amplified and labeled using Cy3-UTP. The labeled cRNAs were then purified using a RNeasy Mini Kit (Qiagen, Hilden, Germany). Fragmentation of 1 μg of labeled cRNA was achieved by adding 11 μl of 10 × (10 ×) blocker and 2.2 μl of 25 × (25 ×) cleavage buffer, heating at 60 °C for 30 min, and diluting the mixture by adding 55 μl of 2 × (2 ×) GE hybrid buffer. Then, 100 μl of hybridization solution was distributed onto gasket slides, which were subsequently assembled onto gene expression microarray slides. The slides were then incubated in an Agilent hybrid oven at 65 °C for 17 h, washed, fixed and scanned using an Agilent DNA microarray scanner. GO enrichment analysis was subsequently performed using the GOSeq R package and DAVID online tools (https://david.ncifcrf.gov/). The analysis of the expression pathways of the differentially expressed genes was performed using the KEGG database (http://www.kegg.jp/kegg/) and KOBAS software.

##### RNA isolation, cDNA synthesis and RT‒qPCR

In accordance with the manufacturer′s instructions, total RNA was extracted from the human NP tissues and cultured human NPCs using TRIzol reagent (Ambion, Life Technologies, Austin, TX, USA) or a miRNeasy Mini Kit (Qiagen, Valencia, CA, USA) to enrich the miRNAs. The quantity and quality of the RNAs were determined using a NanoDrop (Thermo Scientific) and a Bioanalyzer (Agilent Technologies, Santa Clara, CA, USA). cDNA was subsequently prepared using a high-volume cDNA reverse transcription kit containing RNase inhibitors (Applied Biosystems, Foster City, CA, USA) or the miRCURY LNA Universal RT microRNA PCR assay (Exiqon, Woburn, MA, USA). For the miRNAs, RT‒qPCR (Exiqon) was performed using miRCURY LNA SYBR Green Master Mix. Real-time quantitative PCR analysis was then performed using specific primers and SYBR Green PCR Master Mix (Applied Biosystems) for mRNA. U6 snRNA and GAPDH were used as internal reference controls for miRNA and mRNA, respectively. All the reactions were run on a QuantStudio 12 k Flex system (Applied Biosystems) and analyzed using the comparative Ct (ΔΔCt) method (2^−ΔΔCt^ with logarithmic transformation). The specific primers used are listed in Supplementary Table 2.

##### 3′-UTR cloning and luciferase assay

To construct the wild-type FBXW11 3′-UTR reporter plasmid, fragmented FBXW11 3′-UTR gene fragments containing the predicted miR-150-5p binding site were used for PCR amplification. The construct was subsequently cloned and inserted into the psi-CHECKTM-2 vector (Promega, Madison, WI) to firefly DNA downstream of the luciferase gene using XhoI and Noti (Thermo Fisher Science). Site-specific mutations were detected using the QuikChange Lightning site-specific mutation kit, which was subsequently used to produce mutations at the predicted binding site of the wild-type FBXW11 3′-UTR to miR-150-5p (Agilent Technologies, Santa Clara, CA, USA). After PCR amplification, 20 μl of the reactant was digested with DpnI for 1 h at 37 °C, after which 10 μl of the reactant was transferred into DH5 alpha *Escherichia coli* for preparation of the mutant plasmids. All the constructs were confirmed by sequencing (Cosmogenetech, Seoul, Korea). Human NPCs were inoculated into 96-well plates at a density of 3000 cells per well for luciferase experiments. The wild-type or mutant FBXW11 3′-UTR-luciferase reporter plasmids were cotransfected into cells with either the miR-control or miR-150-5p using Lipofectamine PLUSTM reagent, and the cell lysis products were collected after 48 h. Luciferase activity was determined using a Dual-Glo Luciferase Assay System (Promega, Madison, WI, USA) in accordance with the manufacturer’s instructions, and firefly luciferase activity was used as a reference.

##### Flow cytometry, 5-ethynyl-2′-deoxyuridine (EdU) assay and MTT

In accordance with the available instructions, apoptosis was evaluated by labeling cells with Annexin V-FITC and PI using human NPCs. Cells that were positively stained with Annexin V-FITC and negatively stained with PI were considered apoptotic cells. If cells were positively stained with both Annexin V and PI, they were considered necrotic. The cells were stained with 5 µl of Annexin V-FITC and 10 µl of PI for analysis by using EpicsAltra flow cytometry (Beckman Coulter, Brea, CA, USA). For the EdU incorporation assay, human NPCs were seeded into 24-well plates at a density of 2 × 10^5^ per well and cultured at 37 °C in 5% CO_2_. A total of 50 μM EdU (Sigma‒Aldrich) reagent was added to each of the wells, which was maintained for 2 h, followed by fixation with 4% formaldehyde for 15 min and permeation with 0.5% Triton X-100 for 20 min at room temperature. This was followed by elution three times with PBS, after which 100 μl of 1 × Apollo reaction cocktail was added to each well at room temperature for 30 min, after which the cells were stained with Hoechst 33258. The transfection efficiency of the EdU reagent was expressed as the ratio of the number of EdU-positive cells (red cells) to the number of Hoechst 33258-positive cells (blue cells). The MMT test was performed according to the manufacturer′s instructions.

##### Senescence-associated *β*-galactosidase (SA-*β*-gal) staining

Human NPCs were cultivated for 5 h with miR-150-5p mimics, control mimics, miR-150-5p inhibitors, or inhibitor controls. Based on the manufacturer’s instructions, SA-*β*-gal staining was performed using SA-*β*-gal Staining Kit (Beyotime Biotechnology, Shanghai, China). Total cells and SA-*β*-gal positive cells were counted in three random fields per sample. The SA-*β*-gal activity was quantified as the ratio of SA-*β*-gal positive cells (blue) and total cells.

##### Western blot

Human primary NPCs were used to prepare protein lysates in RIPA buffer containing protease and phosphatase inhibitors. A BCA Protein Assay Kit (Thermo Scientific) was used to measure the protein concentrations. The proteins were isolated by using 10% SDS‒PAGE gels and subsequently transferred to PVDF membranes. The PVDF membranes were then incubated with the following primary antibodies at 4 °C in TBS-T containing 5% BSA: Col II (dilution 1:1500, Abcam, product number: ab34712), Aggrecan (dilution 1:1000, Abcam, product number: ab36861), p53 (dilution 1:250, Abcam, product number: ab32049), p21 (dilution 1:2000, Abcam, product number: ab218311), p16 (dilution 1:2000, Abcam, product number: ab51243), MMP3 (dilution 1:500, Abcam, product number: ab52915), MMP9 (dilution 1:2000, Abcam, product number: ab76003), FBXW11 (dilution 1: 2000, Invitrogen, product number: PA5-100524), beta-actin (diluted 1:2000, Abcam, product number: ab8226), and horseradish peroxidase (HRP)-conjugated anti-rabbit IgG (diluted 1:1000, Cell Signaling Technology, product number: 7074). Chemiluminescence was detected using an ECL kit (Amersham Biosciences, Piscataway, NJ, USA) for visualization of immune complexes.

##### Coimmunoprecipitation

Human NPCs were first collected in lysis buffer (25 mM Tris–HCl (pH 7.5), 150 mM NaCl, 0.5% NP-40; protease and phosphatase inhibitors) before centrifugation. The cleaved products were then coimmunoprecipitated using protein agarose beads (G&E Health care, Milwaukee, WI, USA) and primary antibodies. The precipitated protein and the initial whole-cell lysate were then boiled in SDS loading buffer before being separated via SDS‒PAGE and subsequently transferred to PVDF membranes for coincubation with primary and secondary antibodies. The following plasmids were used: pCAG-Myc-IL-6-IP (Addgene, Cambridge, MA, USA), Pad-Track Flag-FBXW11 (Addgene), and Pad-Track Flag-FBXW11 H355A (Addgene).

##### Cell immunofluorescence

Human NPCs were seeded on coverslips in 24-well plates. The sections were subsequently fixed with 4% formaldehyde, 0.5% Triton X-100, 5% BSA sealing solution, primary antibodies, p21 (dilution 1:300, Abcam, product number: ab218311), MMP3 (dilution 1:200, Abcam, product number: ab214794), Alexa Fluor 555 (dilution 1:100, Abcam, product number: ab150078) or Alexa Fluor 488 (dilution 1:1000, Abcam, product number: ab150077), and conjugated secondary antibodies and DAPI (Invitrogen). The level of cellular immunofluorescence was observed under a Carl Zeiss LSM710 confocal microscope (Carl Zeiss, Oberkochen, Germany).

##### Fluorescence in situ hybridization (FISH)

Deparaffinized and permeable sections of the human intervertebral disc were produced using protease K at 37 °C for 10 min, followed by treatment with 3% H_2_O_2_ to inhibit endogenous peroxidase activity and dehydration. Using a 40 nM double-DIG LNA microRNA probe, miR-150-5p (Exiqon, Woburn, MA, USA) was then detected. Then, the cells were hybridized to miR-150-5p at 52 °C for 1 h. The sections were then thoroughly washed with 5 × SSC, 1 × SSC, and 0.2 × SSC buffers and incubated with blocking solution for 15 min, after which the anti-DIG-POD antibody (Roche Applied Science, Indianapolis, IN, USA) was added. According to the manufacturer′s instructions, direct fluorescence detection was performed using the TSA Plus fluorescein system (PerkinElmer, Waltham, MA, USA).

#### Preparation and evaluation of nanoparticle

##### Mass spectrometry analysis

The NP tissues were washed three times with cold PBS and subsequently lysed at 4 °C with 5 ml of hypotonic buffer for 30 min. After centrifugation, the debris was washed three times with 5 mL of hypotonic buffer and subsequently dissolved at 4 °C with 1.5 ml of lysis buffer for 30 min. The resulting supernatant was subsequently incubated with the microbeads, the gene library or EY3 at 4 °C for 1 h. Subsequently, the protein–gene library or protein–EY3 complexes were further incubated with 2 mg (200 μl) of streptavidin beads at 4 °C for 45 min. The denatured protein samples were subsequently resolved via SDS–PAGE followed by staining with Coomassie Brilliant Blue. The purified protein bands of the inducer were then clipped and digested using trypsin. An EASY-nLC 1000 HPLC system (Thermo Scientific) directly connected to a Q Exactive mass spectrometer (Thermo Scientific) was then used for LC‒MS/MS analysis. The analytical column used was an Acclaim PepMap RSLC column (ID 50 μm, length 15 cm, C18, 2 μm, 100A) (Thermo Scientific). Q Exactive mass spectrometry was performed in data-dependent acquisition mode using Xcalibur 2.2 SP1 software and a full-scan mass spectrometer (300–2000 m/z, 70,000 resolution) in an Orbitrap. After 20 data-dependent MS/MS scans at 27% normalized collision energy (HCD) were obtained. The MS/MS spectra from each of the LC–MS/MS runs were subsequently searched against the FASTA files using the SEQUEST HT and Fluorescent 3.0 modules in Proteome Discoverer software (version PD1.4; Thermo Science, USA).

##### Molecular docking and molecular dynamics simulations

Molecular dynamics simulations based on the optimal docking conformation of the FasL-EY3 complex were performed using AMBER16 software. A transition module was used to add hydrogen atoms to initialize the complex, with the default protonation state set to neutral to ionize the amino acids prior to simulation. The AMBER ff99sb force field of the Amber Tools and TIP3P water model were used to generate complex topology files. The neutralization of the complex was achieved by adding Na^+^ at a final salt concentration of 0.15 M. Using the Particle Mesh Ewald (PME) method, molecular dynamics simulations were performed with a minimum distance of 0.1 nm from the edge of the box under periodic boundary conditions. Using the Verlet leapfrog algorithm, the bond lengths were restricted, and the integration time step was set to 2 fs. Afterward, the FasL-EY3 complex was restricted at a harmonic potential force constant of k (Δx)^2^ set to k = 100 kcal/mol^−1^A^−2^. Finally, a 10 ns MD simulation of the protein-aptamer complexes with a time step of 2000 ps was performed at 298 K and 1 atm. The final average structure of the FasL-EY3 complex was determined using 5000 snapshots extracted from the last 10 ns of MD simulation trajectories.

##### Cell-systematic evolution of ligands by exponential enrichment (Cell-SELEX)

The ssDNA library was incubated for 1 h with mouse NPCs at 37 °C. After washing, the bound DNA was eluted and then incubated with nontargeting cells at 37 °C for 0.5–1 h. Following desalination, the supernatant was amplified via PCR. The desired ssDNA was then separated using streptavidin-coated magnetic beads (Promega). Following several rounds of screening, the enriched ssDNA pool was cloned and inserted into the TOP10 chemically competent *Escherichia coli* (TOP10 chemically competent *Escherichia coli*) using a TA cloning kit (Invitrogen) and sequenced. The enriched ssDNA was analyzed in combination with target cells (mouse NPCs) and nontarget cells (mouse chondrocytes and fibroblasts) using a FACScan flow cytometer (BD Immunocytometry Systems, San Jose, CA, USA). The secondary structure was predicted using RNAstructure 5.6 software. The equilibrium dissociation constant (Kd) was calculated using the Formula Y = B_max_ X/(K_d_ + X). The ssDNA library contains a central randomized sequence of 40 nucleotides: 5′-ACTCGCTGA TGACACTACGC-(N)40-AGCATACACGTGCACGTTA T-3′. The dsDNA was synthesized via PCR using a FAM-labeled forward primer (5′-FAM-3′) and a biotin-labeled reverse primer (5′-biotin-3′). The sequence used was 5′-ACTCGCTGATGACACTACGC-3′ and 5′-ATAACGTG CACGTGTATGCT-3′.

##### Nanoparticle synthesis and characterization

A total of 0.6 g of activated polyethylene glycol (CDI-PEG-CDI, Aladdin, Shanghai, China) was dissolved in 14 ml of anhydrous dimethylformamide (DMF), 1.1 g of poly(l-lactide-co-glycolide)-poly(l-lysine) (PLGA-PLL, Sigma, St. Louis, MO, USA) was added, and the mixture was stirred for 48 h under nitrogen protection. The reaction mixture was purified through ultrafiltration using a 7 kDa molecular weight cutoff membrane. Next, methanol dialysis was performed for 24 h, after which any excess unreacted PEG was removed. The resulting product was then vacuum-dried for 24 h to obtain PLGA-PLL-PEG, which was stored at 4 °C for later use. To prepare the 3′-SH-modified aptamer (SH-EY3), 0.1 µmol OH–(CH_2_)6–S–S–(CH_2_)6-aptamer was dissolved in 2.5 ml of 100 mM DTT (dithiothreitol) (pH 8.0) and incubated at room temperature for 30 min. The entire sample was loaded onto a GlenGel-Pak 2.5 (Glen Research, Sterling, VA, USA) desalting column, equilibrated with 25 ml of 50 mM sodium phosphate (pH 6.0), allowed to drip through, and eluted with 2.5 ml of sodium phosphate (pH 6.0). Finally, the purified SH-EY3 conjugate was collected. To bind SH-EY3 to PLGA-PLL-PEG, 1.2 µmol of PLGA-PLL-PEG was dissolved in 5 ml of nuclease-free phosphate buffer solution (pH 7.0) and reacted with 0.6 µmol of SH-EY3 at 4 °C for 12 h. The resulting product, PLGA-PLL-PEG-EY3, was purified by ultrafiltration and stored at 4 °C. To prepare the PLGA-PLL-PEG-EY3/miR-150-5p-Cy5.5 nanoparticles, miRNA-Cy5.5 was mixed with PLGA-PLL-PEG-EY3 at different weight ratios (2:1, 4:1, 8:1, 16:1, 32:1, and 64:1) and then gently vortexed for 30 s. The resulting solution was immobilized for 30 min to form stable PLGA-PLL-PEG-EY3-miR-150-5p-Cy5.5 nanoparticles. An agarose gel retarding assay was used to determine the optimal dose of PLGA-PLL-PEG-EY3 required for complete encapsulation of miRNA. The nanoparticles were characterized by nuclear magnetic resonance hydrogen (1H NMR), dynamic light scattering (DLS) and transmission electron microscopy (TEM).

##### Evaluation of nanoparticle residence time and penetration

The mice were injected with 10 μl of cy5.5, PLGA-PLL-PEG-cy5.5 or PLGA-PLL-PEG-EY3-cy5.5. Images were then taken 15 days, 1 month, 2 months and 3 months after administration using an In Vivo Imaging System (IVIS, Perkin Elmer, Hopkinton, MA, USA) with wave excitation set at 675 nm and a 690–770 nm Cy5.5 filter to determine the fluorescence emission of Cy5.5. After 30 days of in vivo imaging, the mice were euthanized, and their intervertebral discs were removed for permeability analysis. The disc specimens were washed 3 times with HBSS containing antibiotics, followed by treatment with DMEM/F12 (1:1), 10% fetal bovine serum, penicillin/ streptomycin (100 U/0.1 mg/ml), amphotericin B (0.25 μg/ml), and capreomycin (10 μg/ml) overnight at 37 °C and 5% carbon dioxide in six-well plate medium. At the following specified time points, the mouse disc specimens were thoroughly washed and embedded in Tissue-Tek Optimal Cutting Temperature compound (Sakura Finetek USA, Inc., Torrance, CA, USA).

##### Cellular uptake and intracellular distribution

Human NPCs were treated with either PLGA-PLL-PEG-cy5.5 or PLGA-PLL-PEG-EY3-cy5.5 at 37 °C for 6 h, followed by incubation in 50 nM DAPI and 100 nM LysoTracker Green medium at 37 °C for 1 h. The cells were then fixed with 4% paraformaldehyde and viewed under a confocal microscope (FluoView FV1000 confocal microscope, Olympus).

#### In vivo evaluation

##### Aging-related IDD mouse models

To investigate aging-related IDD development, we analyzed the differences in histological phenotypes between C57BL/6 wild-type (WT) and miR-150-5p KO mice at 6, 15, and 22 months of age.

##### Injury-induced IDD mouse models and therapeutic experiments

To establish the injury-induced IDD model as previously described [9, 11], 10-week-old C57BL/6 WT mice were subjected to annulus fibrosus (AF) needle puncture surgery under general anesthesia using an operating microscope. For therapeutic experiments, 20 mice were randomly assigned to four groups. These mice were then intradiscally injected with 10 μl of PLGA-PLL-PEG-EY3/agomiR-150-5p, PLGA-PLL-PEG-EY3/ antagomiR-150-5p or the corresponding negative controls on the 4th, 5th and 6th weeks after IDD surgery. The intervertebral discs in each group were harvested at two time points (at 12 and 18 weeks) for further experiments.

##### X-ray, MRI and histological evaluation

A Faxitron MX20 X-ray machine (Faxitron X-ray Corp., Wheeling, IL, USA) and a 9.4 T MRI scanner (Bruker BioSpec, Germany) were used to scan the mouse tail and vertebrae. Standard 0.5-mm thick T2-weighted spin‒echo coronal scans were obtained with a 0.5-mm spacing between the slices (TR: 5000 ms; TE: 30 ms; FOV: 225 mm; matrix size: 150 × 150). Alcian blue and Alizarin red staining were then performed on the embryonic spine. The epidermis and viscera of the whole embryo were removed, fixed with 95% (v/v) ethanol for 1 week, and then soaked in acetone for 5 days. The specimens were then stained in freshly prepared staining solution containing 70% ethanol in 0.3% Alcian blue (8GX; Sigma‒Aldrich), 95% ethanol in 0.1% alizarin red S, 100% acetic acid, and 100% ethanol at a ratio of 1:1:17. The samples were then destained with 1% KOH for 48 h and 20% glycerin containing 1% KOH for 14 days. Images were then taken using a Zeiss SteREO Discovery V12 microscope. The tissues were embedded in paraffin, sliced and stained with HE, saffron-solid green, or Masson′s and immunohistochemically. Decalcified tissue was then stained with safranin O and scored according to the histological grading criteria.

##### Immunohistochemistry (IHC)

The paraffin-embedded tissue sections of the harvested discs were dewaxed in xylene, dehydrated in an alcohol series, and subsequently rinsed and infiltrated with running water. The sections were then treated at room temperature and incubated for 1 h with primary antibodies against p21 (1:200 dilution; Abcam, product number: ab109520), and MMP3 (1:100 dilution; Abcam, product number: ab52915), followed by secondary antibody labeling and biotin labeling for 30 min. Finally, a VectaStain ABC kit and DAB peroxidase substrate kit (Vector Laboratories, Burlingame, CA, USA) were used for further staining.

##### Immunofluorescence staining

Paraffin-embedded tissue sections of the harvested discs were baked at 60 °C for 15 min in an oven, followed by sequential concentration dehydration with xylene and alcohol. Heat-induced antigen surface repair was performed in a steamer, and the slides were incubated in 10 mM citric acid buffer at 60 °C for 40 min, followed by washing with PBS. The cell membranes were then permeabilized at room temperature for 5 min using 0.1% Triton X-100 in PBS. The following primary antibody was used: Col II (1:300 dilution; Abcam, product number: ab34712), MMP13 (1:100 dilution; Abcam, product number: ab39012). Immune complexes were detected using secondary antibodies.

##### Statistical analysis

GraphPad Prism 8 (GraphPad Software, Inc., La Jolla, CA, USA) was used for all the statistical analyses, and the data are expressed as the means ± standard deviations. For the statistical analysis of the microarray data, a 2-tailed T test adjusted by Benjamin and Hochberg was used. Two-tailed unpaired Student’s t test or the Mann‒Whitney U test was used to compare the differences between the two experimental groups. One-way ANOVA (multiple groups) followed by Tukey′s post hoc analysis was used. A P < 0.05 was considered to indicate statistical significance.

## Results

### Knockout of miR-150-5p potentially relieves intervertebral disc aging and degeneration

**Fig. 1:**
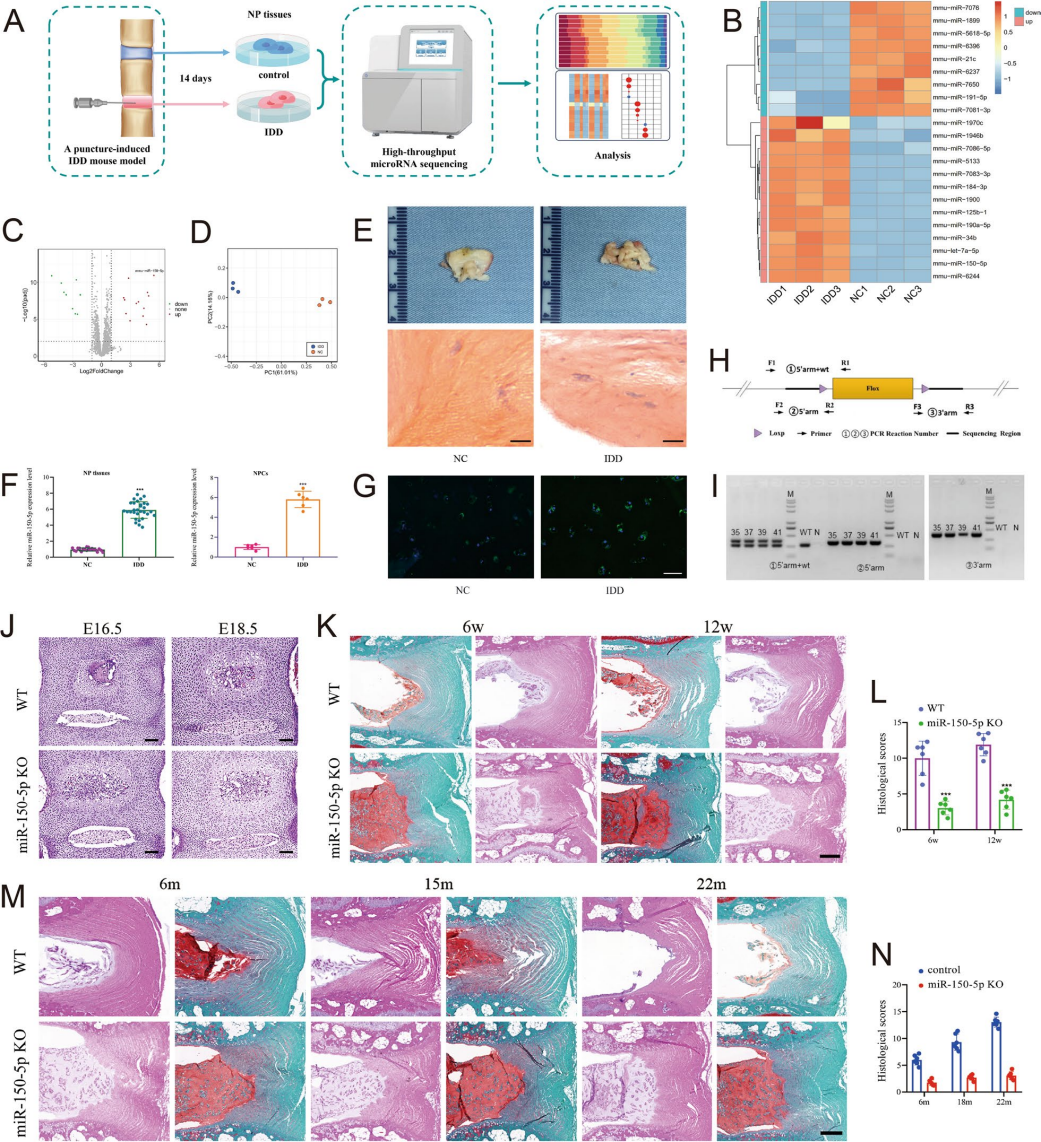
Knockout of miR-150-5p potentially relieves intervertebral disc aging and degeneration. **A** Selection strategy for miRNAs in nucleus pulposus (NP) tissues based on microarray-based sequencing. **B**, **C** Nucleus pulposus cells (NPCs) were obtained from intervertebral discs with and without degeneration (Co6/Co7 vs. Co5/Co6). A heatmap and volcano plot were generated to show the differences in the expression profiles of miRNAs between the intervertebral disc degeneration (IDD) and normal control (NC) groups. There were 22 significantly dysregulated miRNAs in the IDD group (13 miRNAs upregulated and 9 miRNAs downregulated). **D** Principal component analysis (PCA) of miRNA-normalized expression data from NPCs. PCA of the data revealed strong separation between the two groups and good homogeneity within each group for the miRNA arrays. **E** Histological examination of NP tissues from NCs and IDD patients. (Scale bar, 100 μm.) **F** Compared with those in controls (n = 30), miR-150-5p expression levels were increased in NP cells and tissues from IDD patients (n = 67). ***P < 0.001 by the Mann‒Whitney U test. **G** FISH analysis demonstrated that the levels of miR-150-5p were greater in the NPCs of IDD patients than in those of controls. (Scale bar, 50 μm.) **H** Targeting strategy for generating miR-150-5p knockout (KO) mice. **I** The genotypes of the miR-150-5p KO mice were confirmed by Southern blot analysis. **J** Intervertebral discs were harvested from wild-type (WT) and miR-150-5p KO littermate embryos (E16.5 and E18.5). Hematoxylin and eosin (HE) staining was used to visualize the intervertebral discs of WT and miR-150-5p KO littermate embryos (E16.5 and E18.5). The development of intervertebral discs was not significantly different between WT and miR-150-5p KO littermates. (Scale bar, 100 μm.) **K** WT and miR-150-5p KO littermates were subjected to needle puncture-induced IDD surgery. Representative images of 6- and 12-week post-IDD surgery intervertebral disc sections. (Scale bar, 100 μm.) **L** Histological score indicating that miR-150-5p KO could attenuate IDD development (n = 6 per group). P < 0.01 was determined by two-tailed unpaired Student′s t test. **M** HE and Safranin O staining of intervertebral discs from WT or miR-150-5p KO mice at 6, 15 and 22 months. (Scale bar, 100 μm.) **N** Histological analysis indicated that miR-150-5p KO could ameliorate age-related IDD in mice. (n = 6 per group) P < 0.01 by two-tailed unpaired Student’s t test

An injury-induced IDD mouse model was established via needle puncture as previously described [9, 11]. The coccygeal discs of Co6/Co7 were exposed and then punctured with a 34G syringe needle through the annulus fibrosus (AF) to the nucleus pulposus (NP). The needle was then left in the intervertebral disc at a depth of 1.5 mm for a period of 10 s. The adjacent Co5/Co6 disc levels, which were used as contrast segments, were not punctured. Intervertebral discs were collected 14 days after needle puncture, and the differentially expressed miRNAs were subsequently screened via a miRNA microarray chip (Affymetrix® 4.0 miRNA Array, USA) (Fig. 1A).

NPCs were obtained from intervertebral discs with and without degeneration (Co6/Co7 vs*.* Co5/Co6). A heatmap and volcano plot were generated to show the expression profiles of the miRNAs, which included 22 significantly dysregulated miRNAs in the IDD group (13 upregulated miRNAs and 9 downregulated miRNAs) (Fig. 1B, C). PCA of the data revealed strong separation between the two groups and good homogeneity within each group for the miRNA arrays (Fig. 1D). Only miRNAs with a mean fold change > 5.0 or < 0.10 and a p < 0.01 were selected for further analysis. According to the abovementioned criteria, miR-150-5p, miR-6244, miR-125b-1, miR-1946b, miR-1899 and miR-21c were significantly dysregulated (Supplementary Table 3). These miRNAs were further evaluated by RT‒qPCR and FISH using an additional independent cohort comprising 67 IDD patients and 30 controls (Fig. 1E–G). Among these miRNAs, miR-150-5p was found to be one of the most significantly upregulated in IDD patients compared with controls.

miR-150-5p has been shown to play a critical role in OA development [35, 36]. As chondrocytes are extremely similar to NPCs [37], we selected miR-150-5p for further investigation. miR-150-5p KO mice were purchased from Jackson Laboratories (Bar Harbor, ME, USA). The targeting strategy used to generate miR-150-5p KO mice is described in Fig. 1H and was confirmed by Southern blotting (Fig. 1I). Pregnant mice with embryos at E12.5 were injected with tamoxifen (100 μg/g body weight; Sigma, St. Louis, MO, USA). Hematoxylin and eosin (HE) staining of spines was performed for E16.5 and E18.5 embryos (Fig. 1J), and no significant differences were detected between wild-type and miR-150-5p KO littermates. The results indicated that miR-150-5p deficiency had no obvious effects on the embryonic intervertebral disc development.

To explore the role of miR-150-5p in IDD, we subjected 8-week-old WT and miR-150-5p KO mice to needle puncture-induced IDD surgery or sham surgery. At 6 and 12 weeks after IDD surgery, HE staining of the intervertebral disc showed fewer degenerated NPs and better organized AF tissues in the miR-150-5p KO mice. However, control mice exhibited severe disruption of the NP and AF structures (Fig. 1K, L). These findings suggested that miR-150-5p KO could attenuate IDD development.

To further investigate whether miR-150-5p deficiency affects IDD in aging mice, we observed spontaneous disc degeneration in miR-150-5p KO mice with aging. HE staining of intervertebral discs was performed for WT and miR-150-5p KO mice at 6, 15 and 22 months. Histological analysis of postnatal 6-month-old WT and miR-150-5p KO mice indicated intact outer AF and inner NP (Fig. 1M, N). By postnatal month 6 and 15, mild and moderate disc degeneration was noted in the WT mice. By postnatal month 15, the IDD-like features of the WT mice were aggravated compared with those of the miR-150-5p KO mice. Taken together, these results indicated that miR-150-5p KO could ameliorate aging-related IDD in mice.

### Downregulation of miR-150-5p attenuates NPC senescence

**Fig. 2:**
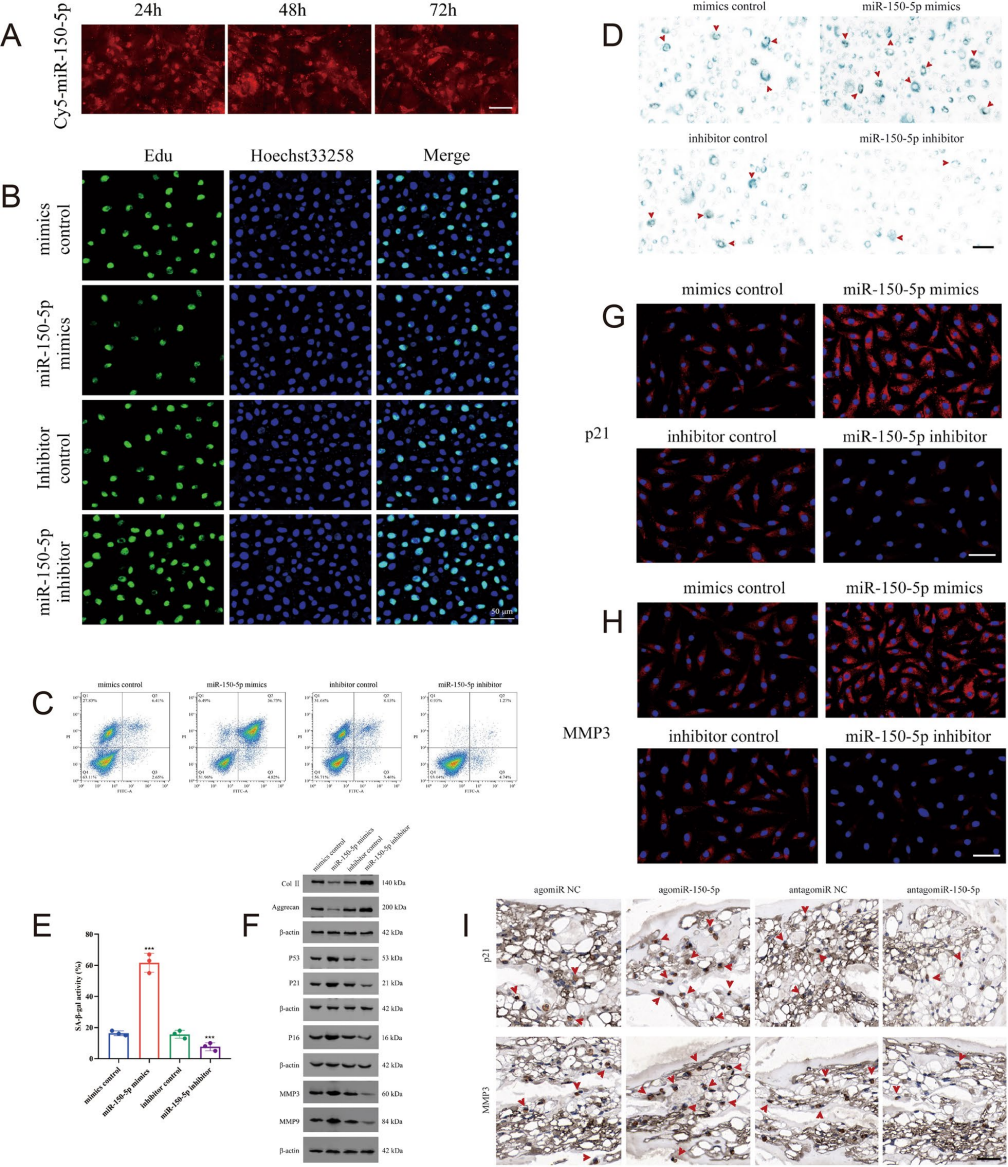
Downregulation of miR-150-5p attenuates nucleus pulposus cell senescence. **A** Cy5-miR-150-5p was taken up by nucleus pulposus cells (NPCs) at 24, 48, and 72 h. (Scale bar, 20 μm.) **B** EdU assays showed that the level of cellular proliferation was increased in human NPCs transfected with the miR-150-5p inhibitor. (Scale bar, 50 μm.) **C** Flow cytometry showed that the apoptosis rate was decreased in human NPCs transfected with the miR-150-5p inhibitor. **D** Senescence-associated *β*-galactosidase (SA-*β*-gal) staining demonstrating that cellular senescence was decreased in human NPCs transfected with the miR-150-5p inhibitor. (Scale bar, 20 μm.) **E** Bar graphs showing the quantification of the relative number of SA-*β*-gal-positive cells (n = 3). **F** Western blot analysis showed that the expression levels of P53, P21, P16, MMP3 and MMP9 were affected by the upregulation or downregulation of miR-150-5p. **G**, **H** Immunofluorescence analysis showed that the expression levels of P21 and MMP3 were downregulated in human NPCs transfected with the miR-150-5p inhibitor. (Scale bar, 20 μm.) **I** Intradiscal injection of miR-150-5p mimics, mimic control, miR-150-5p inhibitor, or control in the needle puncture-induced IDD mouse model. The disc tissues were then harvested at the 12th week after IDD surgery. Immunohistochemistry showed the P21 and MMP3 expression levels in the four groups. (Scale bar, 100 μm.)

To explore the effect of miR-150-5p on IDD in vitro, a miR-150-5p inhibitor or mimic was transfected into primary human NPCs. The load efficacy was detected by evaluating the cellular uptake of Cy5-labeled fluorescence. The fluorescent signal (red) showed the efficient transfection of miR-150-5p into NPCs (Fig. 2A). EdU assays showed that the miR-150-5p inhibitor exerted a positive effect on NPC proliferation, in contrast to the effect of the miR-150-5p mimics (Fig. 2B). The suppressive influence of the miR-150-5p inhibitor on NPC apoptosis was confirmed by flow cytometry (Fig. 2C). Senescence-associated *β*-galactosidase (SA-*β*-gal) staining revealed a decrease in the number of SA-*β*-gal-positive NPCs in the miR-150-5p inhibitor group, indicating that downregulated miR-150-5p may delay NPC senescence (Fig. 2D, E).

Functional experiments were used to detect the effects of miR-150-5p expression on the expression of cellular senescence markers, the senescence-associated secretory phenotype (SASP) and anabolism/catabolism. Western blot analysis of the miR-150-5p inhibitor group revealed downregulated expression of cellular senescence markers (P53, P21 and P16) and SASP markers (MMP3 and MMP9) and upregulated expression of anabolic/catabolic markers (Col II and Aggrecan) (Fig. 2F, Supplementary Fig. 1). These changes were further verified using immunofluorescence (Fig. 2G, H).

Intradiscal injection of miR-150-5p mimics, mimic control, miR-150-5p inhibitor, or inhibitor control in mouse models was performed 4, 5 and 6 weeks after needle puncture surgery. The intervertebral disc tissues were then harvested at 12 weeks after the needle puncture surgery. IHC analysis revealed that cellular senescence markers and SASP markers (P21 and MMP3) were expressed at low levels in IDD mice treated with the miR-150-5p inhibitor. In contrast, high expression levels of P21 and MMP3 were found in the miR-150-5p mimic group (Fig. 2I). Our results revealed that the inhibition of miR-150-5p alleviated NPC senescence.

### FBXW11 can potentially regulate the targeting of miR-150-5p

**Fig. 3:**
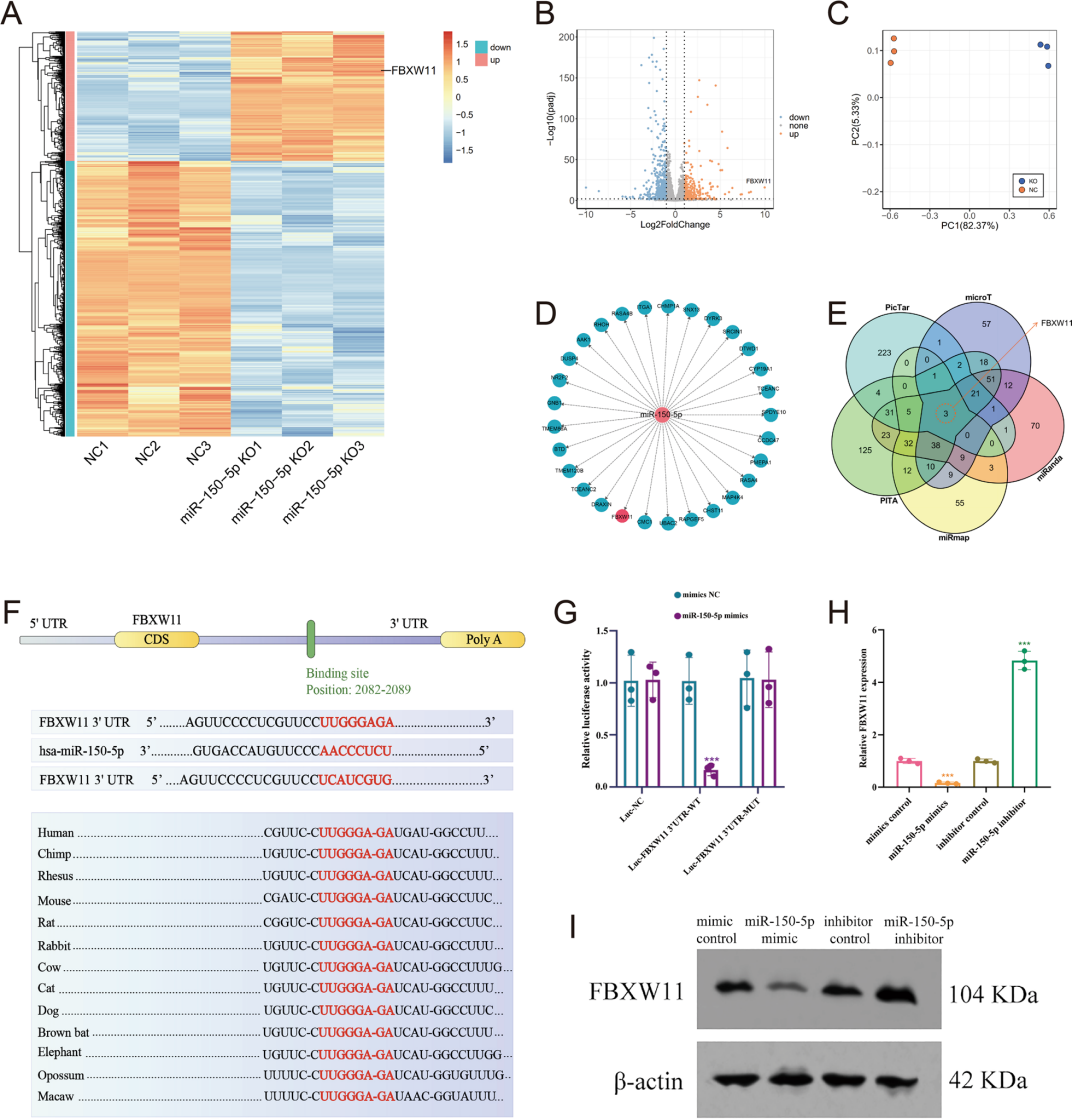
FBXW11 can potentially regulate the targeting of miR-150-5p. **A**–**C** Nucleus pulposus cells (NPCs) were harvested from wild-type (WT) mice (n = 3) and miR-150-5p knockout (KO) mice (n = 3). RNA sequencing of NPCs indicated that FBXW11 is a potential regulatory target of miR-150-5p. A heatmap (**A**) and volcano plot (**B**) showing the genes differentially expressed between the NPCs of the WT and miR-150-5p KO mice. **C** The PCA of the data revealed strong separation between the two groups and good homogeneity within each group for mRNA arrays. **D**, **E** Bioinformatics data showing that miR-150-5p was computationally predicted to target FBXW11 by different algorithms. **F** The 3′-UTR of FBXW11 mRNA contains potential binding sites for miR-150-5p. This sequence alignment showed a high level of sequence conservation and complementarity with miR-150-5p. **G** Relative luciferase activity detection in human NPCs cotransfected with miR-150-5p mimic/inhibitor and FBXW11 3′-UTR-wild/mutant-type reporter plasmids. **H**, **I** The qRT‒PCR and Western blot results showed that the FBXW11 mRNA and protein expression levels were decreased and increased, respectively, in human NPCs following transfection with miR-150-5p mimics and inhibitors

NPCs were harvested from WT (n = 3) and miR-150-5p KO mice (n = 3). RNA sequencing of NPCs revealed 126 genes differentially expressed between the control and miR-150-5p KO NPCs. FBXW11 was one of most significantly downregulated genes (Fig. 3A–C). miR-150-5p was computationally predicted to target FBXW11 by different algorithms (Fig. 3D, E). Bioinformatic analysis revealed sequence alignment of a putative miR-150-5p-binding site within the 3′-UTR of FBXW11 mRNA (Fig. 3F). A direct link between miR-150-5p and FBXW11 was confirmed by a luciferase reporter assay (Fig. 3G). Furthermore, RT‒qPCR and Western blot analysis revealed that FBXW11 expression was decreased in the miR-150-5p mimic group but was increased in the miR-150-5p inhibitor group (Fig. 3H, I). These results indicated that FBXW11 might be a potential regulatory target of miR-150-5p.

### The effects of miR-150-5p on the FBXW11/TAK1/NF-κB pathway

**Fig. 4:**
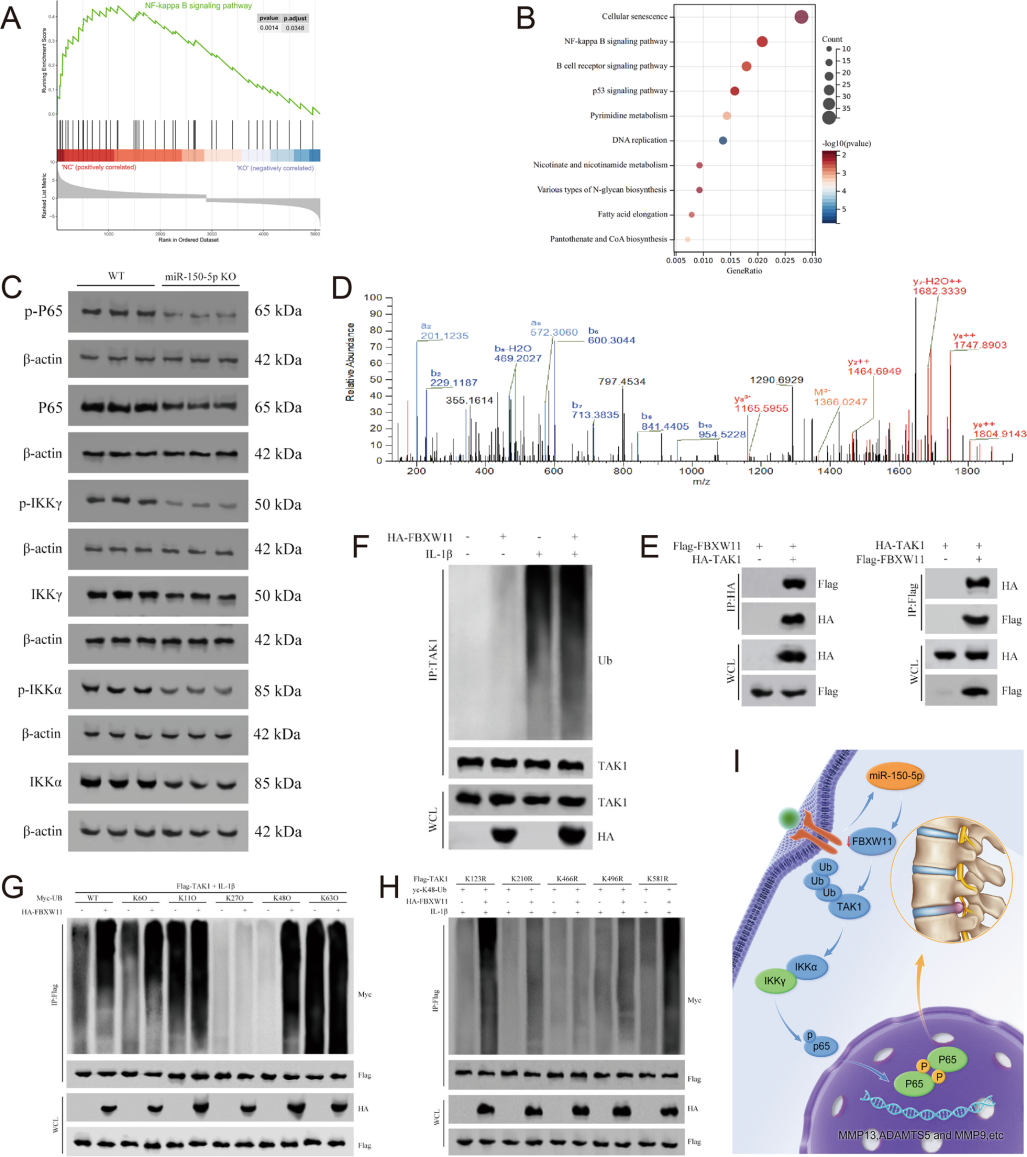
The effects of miR-150-5p on the FBXW11/TAK1/NF-κB pathway. **A**, **B** The NF-κB signaling pathway was predicted to be the pathway most enriched with upregulated miRNAs by KEGG analysis. **C** The expression levels of proteins in the NF-kB signaling pathway, including p-P65, P65, p-IKKγ, IKKγ, p-IKKα and IKKα, in nucleus pulposus cells (NPCs) from wild-type and miR-150-5p knockout (KO) mice. **D** TAK1 was identified as a molecular target that interacts with FBXW11 by mass spectrometry (LC‒MS/MS). **E** Coimmunoprecipitation (co-IP) indicated that FBXW11 may directly interact with TAK1. **F**–**H** FBXW11 negatively regulated TAK1 through K48-linked ubiquitination at the K446 residue. **I** The FBXW11/TAK1/NF-κB pathway may be the fundamental mechanism underlying the progression of IDD induced by miR-150-5p

KEGG enrichment analysis revealed that the NF-κB signaling pathway was the pathway associated with the most upregulated miRNAs (Fig. 4A, B). Using WT and miR-150-5p KO mice, we used intervertebral disc tissues to verify the association between miR-150-5p and the NF-κB signaling pathway. Western blot analysis revealed that key proteins involved in the NF-κB signaling pathway, including P65, p-P65, IKKγ, p-IKKγ, IKKα and p-IKKα, were dysregulated in miR-150-5p KO mice (Fig. 4C). FBXW11, as the target of miR-150-5p, plays a critical role in regulating protein activity through the ubiquitination of phosphorylated substrates [38, 39]. Liquid chromatography with tandem mass spectrometry (LC‒MS/MS) and coimmuno-precipitation (co-IP) indicated that FBXW11 may directly interact with TAK1 (Fig. 4D, E). Importantly, we further found that FBXW11 negatively regulated TAK1 through K48-linked ubiquitination at the K446 residue (Fig. 4F–H). Our findings indicate that the FBXW11/TAK1/NF-κB pathway may be the fundamental mechanism underlying the progression of IDD induced by miR-150-5p (Fig. 4I).

### Identification of the binding proteins and selection of NPC-specific aptamers

**Fig. 5:**
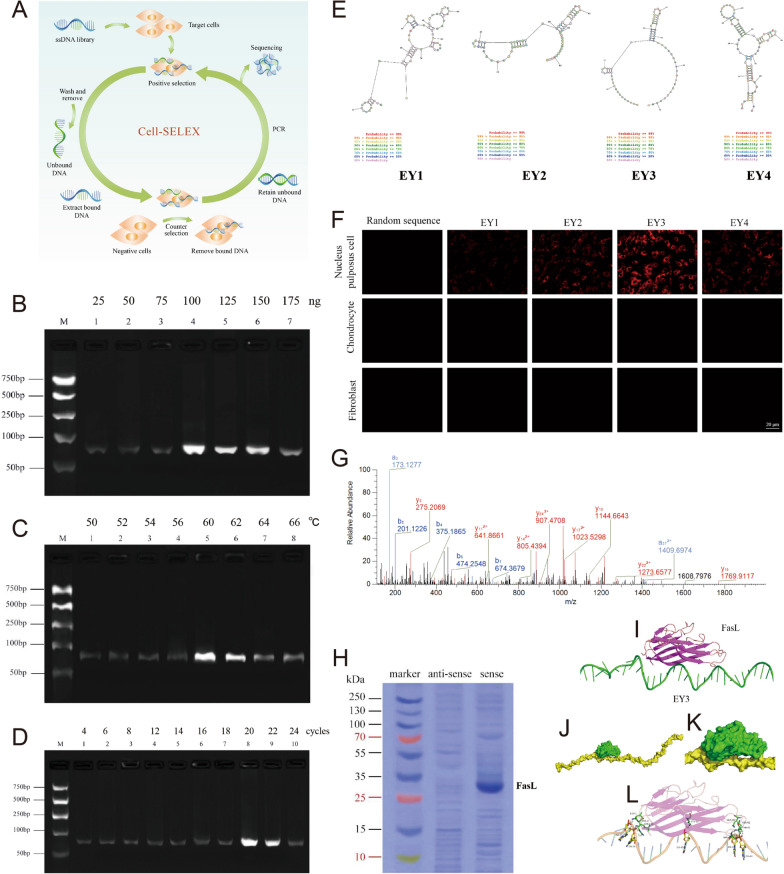
Identification of the binding proteins and selection of nucleus pulposus cell-specific aptamers. **A** Schematic illustration of aptamer selection in nucleus pulposus cells (NPCs). **B**–**D** Essential strategies for optimizing PCR conditions to generate ssDNA aptamers in SELEX. The highest proportion of DNA of interest relative to unwanted products was considered the optimum condition. The results revealed that the best values for final template concentration (**B**), annealing temperature (**C**) and number of PCR cycles (**D**) were 100 ng, 60 °C and 20 cycles, respectively. **E** Proposed secondary structure of the EY1-4 aptamer. **F** The subcellular localization of EY3 in NPCs, chondrocytes and fibroblasts. (Scale bar, 20 μm.) **G** Annotated MS/MS spectrum assigned to the FasL peptide. The data were acquired from the analysis of the samples by high-sensitivity LC‒MS/MS on a Q Exactive mass spectrometer. **H** Coomassie blue-stained SDS‒PAGE was used to analyze aptamer-assisted target purification. **I**–**L** Model of the interaction between EY3 and FasL.

The newly developed cell-based systematic evolution of ligands by exponential enrichment (SELEX) technique was employed to select NPC-specific aptamers for miRNA-based therapeutics (Fig. 5A) [12, 16]. Asymmetric PCR optimization was used to generate ssDNA aptamers in SELEX. The highest proportion of DNA of interest relative to unwanted products was considered the optimum condition. The results of agarose gel analysis demonstrated that the best values for the final template concentration, annealing temperature and number of PCR cycles were 100 ng, 60 °C and 20 cycles, respectively (Fig. 5B–D). We selected EY3 as the optimal aptamer among the EY1-4 aptamer candidates (Fig. 5E, Supplementary Fig. 2). The secondary structure of the EY3 aptamer was more stable and cost-effective than that of the other aptamers and may lead to binding to nanoparticles. Flow cytometry analysis revealed that EY3 had a high NPC-specific conjugation efficiency (Supplementary Fig. 3). The specific binding capacity of EY3 to NPCs was examined by immunofluorescence using fluorescein isothiocyanate (FITC) as a stain (Fig. 5F).

To detect the binding of the target molecules to EY3, NPCs were treated with proteinase K for 60 min, followed by incubation with EY3 for 30 min. This step involves identifying the membrane proteins to which EY3 is attached. Total membrane proteins were isolated from NPCs, which were subsequently treated with either biotinylated EY3 or the library. LC‒MS/MS analysis revealed that FasL had the highest score and the highest score among the potential candidates (Fig. 5G). FasL was confirmed to be a target of EY3 by Coomassie blue-stained SDS‒PAGE (Fig. 5H). Structural analysis was subsequently performed to reveal the molecular basis for the dynamic interactions of the FasL-EY3 complexes (Fig. 5I–K). Furthermore, molecular docking analysis revealed that several amino acids form crucial interactions with EY3 and FasL (Fig. 5L).

### The PLGA-PLL-PEG-EY3 nanoparticles facilitate miR-150-5p delivery into NPCs

**Fig. 6:**
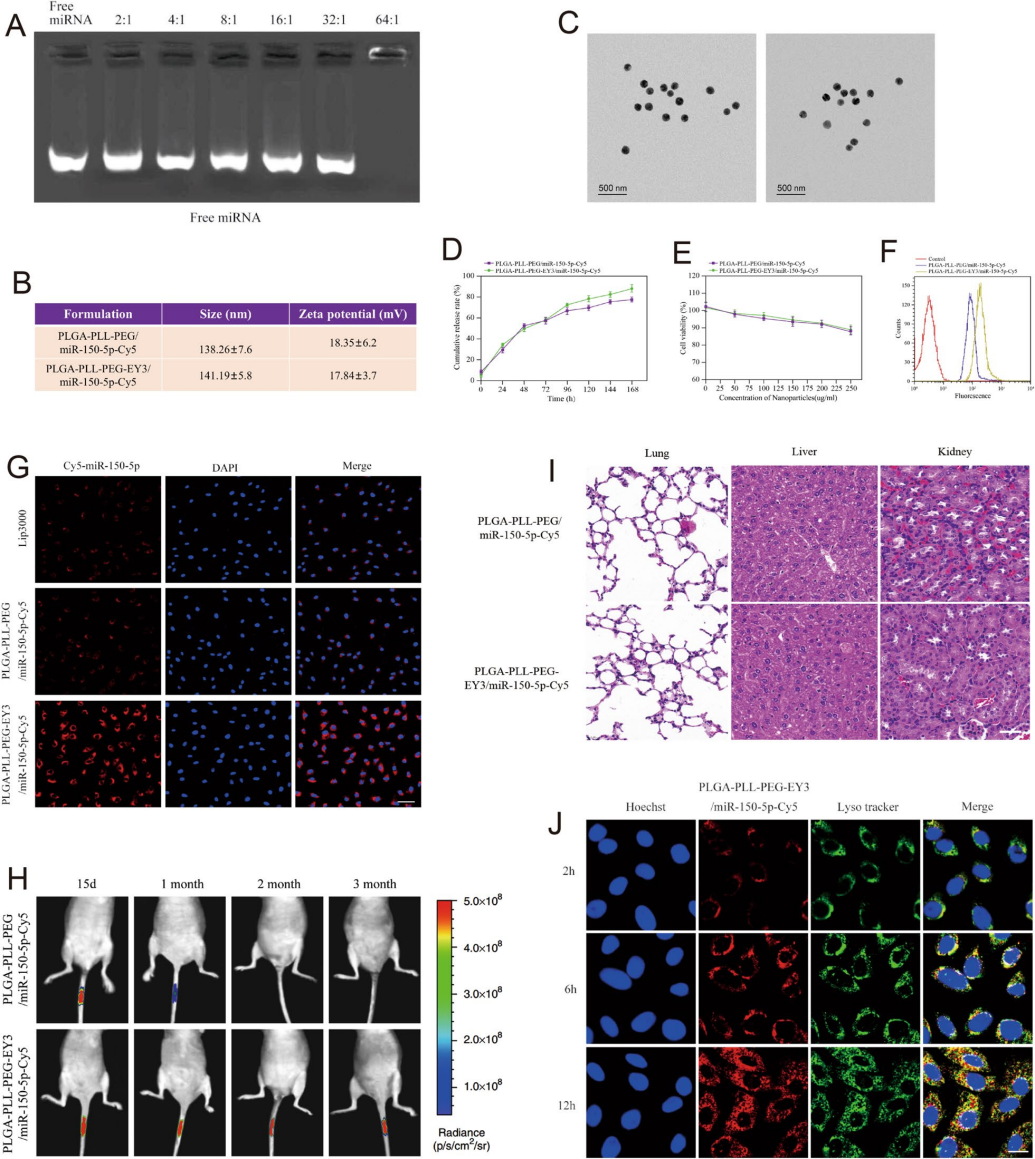
The PLGA-PLL-PEG-EY3 nanoparticles facilitate miR-150-5p delivery into nucleus pulposus cells. **A** Electrophoretic mobility of miRNAs in an agarose gel. **B** The surface zeta potential and diameter of PLGA-PLL-PEG/miR-150-5p-Cy5 with or without an aptamer (EY3). **C** Transmission electron microscopy (TEM) image of the PLGA-PLL-PEG/miR-150-5p-Cy5 nanoplatform with or without an aptamer (EY3). (Left: PLGA-PLL-PEG/miR-150-5p-Cy5; Right: PLGA-PLL-PEG-EY3/miR-150-5p-Cy5) (scale bar, 500 nm). **D** Release profile of PLGA-PLL-PEG/miR-150-5p-Cy5 and PLGA-PLL-PEG-EY3/miR-150-5p-Cy5 within 168 h. Drug release analysis revealed the controlled release of PLGA-PLL-PEG-EY3/miR-150-5p-Cy5, for which approximately 70% of the material was released after 96 h. **E** In vitro viability of nucleus pulposus cells (NPCs) treated with PLGA-PLL-PEG/miR-150-5p-Cy5 and PLGA-PLL-PEG-EY3/miR-150-5p-Cy5. **F** Flow cytometry image of the PLGA-PLL-PEG/miR-150-5p-Cy5 and PLGA-PLL-PEG-EY3/miR-150-5p-Cy5 binding peaks. **G** Cellular uptake of PLGA-PLL-PEG/miR-150-5p-Cy5 with or without an aptamer (EY3) after 8 h of incubation. (Scale bar, 100 μm.) **H** In vivo fluorescence imaging of intervertebral discs 3 months after intradiscal injection of PLGA-PLL-PEG/miR-150-5p-Cy5 or PLGA-PLL-PEG-EY3/miR-150-5p-Cy5. (Scale bar, 100 μm.) **I** Hematoxylin and eosin (HE) staining of the lungs, livers and kidneys of mice 3 months after intradiscal injection of PLGA-PLL-PEG/miR-150-5p-Cy5 or PLGA-PLL-PEG-EY3/miR-150-5p-Cy5. (Scale bar, 20 μm.) **J** Intracellular distribution of PLGA-PLL-PEG-EY3/miR-150-5p-Cy5 in nucleus pulposus cells. (Scale bar, 20 μm.)

A novel PLGA-PLL-PEG-EY3 nanoparticle was constructed to deliver miR-150-5p to NPCs to rescue IDD. To test the stability of the nanoparticles, we found that miR-150-5p-Cy5.5 mixed with PLGA-PLL-PEG-EY3 at a 64:1 weight ratio could completely encapsulate the miRNA molecules (Fig. 6A). Subsequently, we examined the size and zeta potential of the nanoparticles. Dynamic light scattering (DLS) confirmed the similarity and consistency of the differences in nanoparticle size and zeta potential between PLGA-PLL-PEG/miR-150-5p-Cy5 and PLGA-PLL-PEG-EY3/miR-150-5p-Cy5, indicating that the EY3 aptamer had no significant effect on the structure of the nanoparticles (Fig. 6B). The nanoparticles were also detected by transmission electron microscopy (TEM) to confirm that they were spherical (Fig. 6C). In accordance with the results of DLS, nanoparticle loading with the EY3 aptamer did not induce structural changes in the TEM images. Furthermore, we observed the release profiles of PLGA-PLL-PEG/miR-150-5p-Cy5 and PLGA-PLL-PEG-EY3/miR-150-5p-Cy5 within 168 h. Drug release analysis revealed the controlled release of PLGA-PLL-PEG-EY3/miR-150-5p-Cy5, for which the release rate was approximately 70% after 96 h (Fig. 6D). To evaluate cell viability, primary NPCs were incubated with various concentrations (0 ~ 250 μg/ml) of the two types of nanoparticles for 24 h. Cell viability analysis indicated that there was little effect on cell proliferation in NPCs exposed to PLGA-PLL-PEG/miR-150-5p-Cy5 or PLGA-PLL-PEG-EY3/miR-150-5p-Cy5 (Fig. 6E). The results showed no toxicity in NPCs treated with the two types of nanoparticles. Our flow cytometry results revealed that cells treated with PLGA-PLL-PEG-EY3/miR-150-5p-Cy5 nanoparticles had significantly greater fluorescence intensities than did the other groups (Fig. 6F), demonstrating greater affinity for nanoparticle-NPC binding. Immunofluorescence staining revealed an increase in the density of cellular uptake in NPCs treated with PLGA-PLL-PEG-EY3/miR-150-5p-Cy5 (Fig. 6G). This suggested that PLGA-PLL-PEG-EY3/miR-150-5p-Cy5 could enhance the transfection efficiency of NPCs. In vivo fluorescence imaging revealed that mice intradiscal injected with PLGA-PLL-PEG-EY3/miR-150-5p-Cy5 maintained a sustained fluorescence signal for 3 months. In comparison, an intradiscal injection of PLGA-PLL-PEG/miR-150-5p-Cy5 into mouse intervertebral discs resulted in a transient peak in the fluorescence signal at 0.5 or 1 month. These results demonstrated that, compared to PLGA-PLL-PEG nanoparticles, PLGA-PLL-PEG-EY3 nanoparticles may be the predominant effective NPC-targeted delivery agent for miR-150-5p in vivo (Fig. 6H).

The in vivo toxicity of the nanoparticles was investigated by histological analysis of the major organs of the mice injected with the nanoparticles. No pathological abnormalities were observed in the liver, lungs, or kidneys (Fig. 6I). Endosomal entrapment may be the main biological barrier for nanoparticle-mediated intracellular delivery of miRNAs. In this step, we used Cy-5, LysoTracker Green, and Hoechst to label miRNAs, lysosomes, and cell nuclei, respectively. Images were obtained at 2, 6, and 10 h post transfection. Immunofluorescence staining confirmed that the nanoparticles could successfully escape from the endosome to reach the cytosol (Fig. 6J).

### Intradiscal delivery of miR-150-5p inhibitor by the PLGA-PLL-PEG-EY3 nanoparticles alleviates disc degeneration in an IDD mouse model

**Fig. 7:**
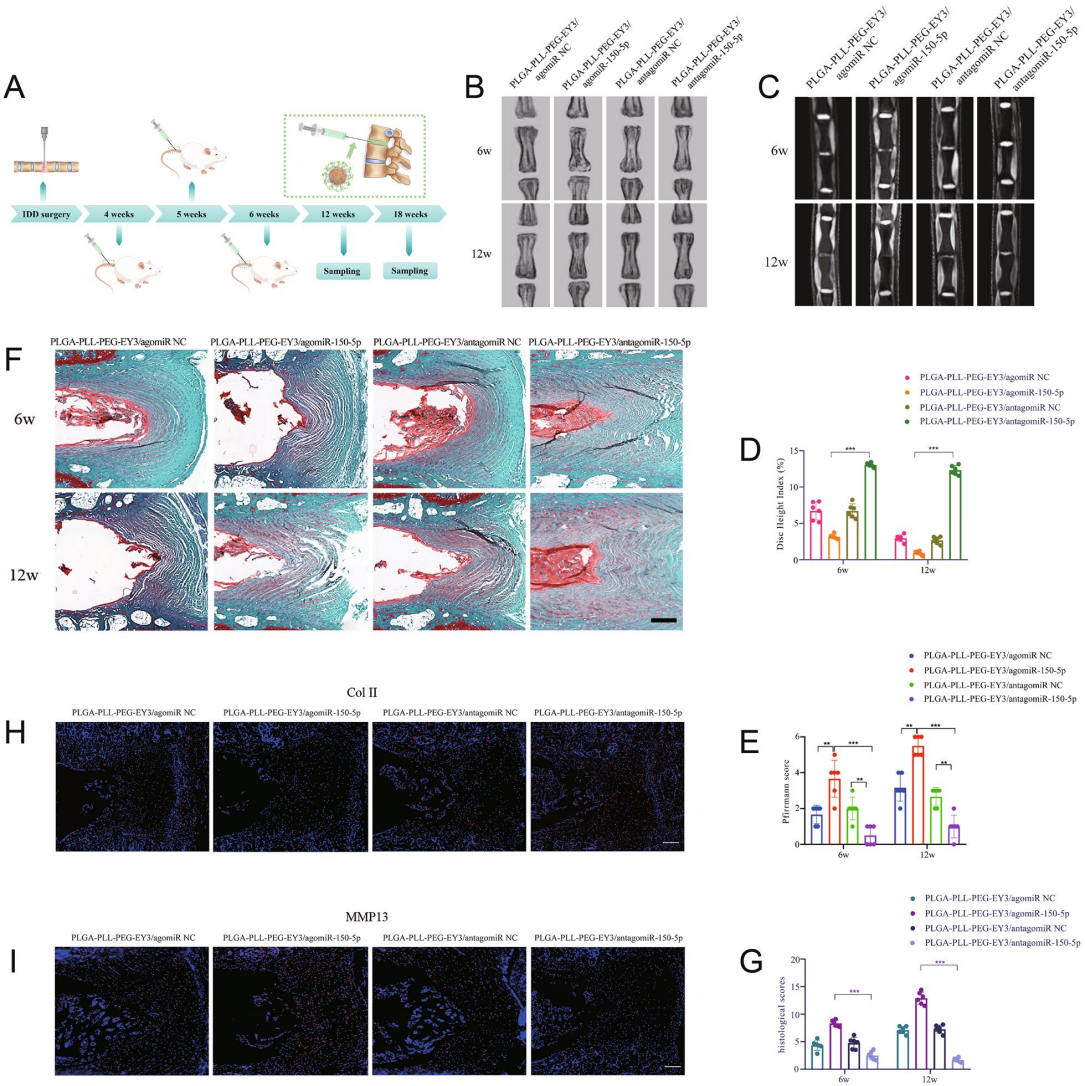
Intradiscal delivery of miR-150-5p inhibitor by the PLGA-PLL-PEG-EY3 nanoparticles alleviates intervertebral disc degeneration in a mouse model. **A** Schematic representation of the experimental design. **B**, **C** X-ray and MRI images showing an alleviation of intervertebral space collapse in mice treated with PLGA-PLL-PEG-EY3/antagomiR-150-5p (6 weeks, 12 weeks post intradiscal injection). **D**, **E** The disc height index and Pfirrmann score were significantly lower in mice treated with PLGA-PLL-PEG-EY3/antagomiR-150-5p (6 weeks and 12 weeks after intradiscal injection). **F** Safranin-O staining images of intervertebral discs from the different treatment groups. **G** Histological scores were significantly lower in mice treated with PLGA-PLL-PEG-EY3/antagomiR-150-5p (6 weeks, 12 weeks after intradiscal injection). **H**, **I** Immunohistochemistry assays showed increased Col II and decreased MMP13 expression in mice treated with PLGA-PLL-PEG-EY3/antagomiR-150-5p

Finally, we explored the effect of nanoparticles/miR-150-5p therapeutics in IDD mice by radiographic and histological assessments. Considering the possible future clinical applications of these nanoparticles, we evaluated their ability to block IDD progression 4 weeks after needle puncture surgery. We believe that these findings closely simulate the most common conditions and symptoms clinically presented by most patients with IDD (Supplementary Fig. 4). Briefly, the mice underwent needle puncture surgery to mimic IDD phenotypes in vivo. After 4 weeks, nanoparticles (PLGA-PLL-PEG-EY3/agomiR-150-5p, PLGA-PLL-PEG-EY3/antagomiR-150-5p and corresponding negative controls) were intradiscally injected into IDD mice once every week for three consecutive weeks. At two time points (6 and 12 weeks after intradiscal injection), the mice were evaluated by X-ray and MRI analysis and then sacrificed. Intervertebral disc tissue was dissected for Safranin-O staining and immunofluorescence (Fig. 7A).

As shown in Fig. 7B–E, the coccygeal disc Co6/Co7 was the experimental location, and the adjacent Co5/Co6 disc was the contrast segment. The IDD-induced surgical procedure caused significant radiographic changes in the disc, including (1) a narrow disc space and (2) darkening of the disc (black disc) on MRI, revealing aggravated disc degradation as a sign of IDD symptoms. Based on the X-ray and MRI images (Fig. 7B, C), a narrow disc space and black disc were present in the PLGA-PLL-PEG-EY3/agomiR-150-5p group and two control groups. In contrast, the Co6/Co7 disc height and white signal intensity were relatively normal in the PLGA-PLL-PEG-EY3/antagomiR-150-5p treatment group, which was almost identical to those of the adjacent Co5/Co6 disc (healthy control). Dynamic changes in the X-ray and MRI images were confirmed by the disc height index and Pfirrmann score (Fig. 7D, E). This finding suggested that IDD progression was obviously alleviated by PLGA-PLL-PEG-EY3/antagomiR-150-5p. The results of Alcian blue staining support the above conclusion. Severe destruction of the NP and collapse of the AF were observed in IDD mice injected with PLGA-PLL-PEG-EY3/agomiR-150-5p (Fig. 7F). However, the phenotype in the PLGA-PLL-PEG-EY3/antagomiR-150-5p treatment group showed minimal NP and AF degradation in the discs. The severity of IDD lesions was evaluated by two independent investigators according to the histopathological scoring system. This blinded assessment showed that the intradiscal delivery of PLGA-PLL-PEG-EY3/antagomiR-150-5p had the lowest histological score, indicating successful NP and AF repair in IDD model mice (Fig. 7G). Disc tissues were collected to examine the expression of MMP13 and Collagen II. PLGA-PLL-PEG-EY3/antagomiR-150-5p treatment led to reduced MMP13 expression and increased Collagen II expression in disc tissue via intradiscal injection (Fig. 7H, I). Taken together, our findings indicated that PLGA-PLL-PEG-EY3 carrying antagomiR-150-5p (a miR-150-5p inhibitor) could protect against NP degradation and ameliorate IDD development.

## Discussion

In the present study, via high-throughput microarray analysis, we first found that the expression level of miR-150-5p was increased in a needle puncture-induced IDD mouse model. Further validation was performed in IDD patient and miR-150-5p KO mouse samples. Gain- and loss-of-function research has shown that overexpression of miR-150-5p impairs the balance between ECM anabolism and catabolism, whereas silencing of miR-150-5p attenuates NPC senescence and ECM destruction. Our in vivo experiments suggested that the IDD phenotype was induced by aging and needle puncture surgery and was alleviated by miR-150-5p KO. In a preclinical study, we explored whether nanoparticles loaded with miR-150-5p may be promising therapeutics for inhibiting the senescence of NPCs, regulating ECM homeostasis, and rescuing disc degeneration.

NPC senescence is thought to be one of the molecular mechanisms involved in age-related IDD [40, 41]. Accumulating evidence suggests that cellular senescence in NPs promotes IDD aggravation, which can be regulated by miRNAs [3, 30, 42, 43]. In miR-150-5p KO mice, we observed downregulated levels of senescence-associated markers, upregulated levels of the targeted gene FBXW11, and dysregulated levels of the NF-κB transcription factor. Mechanistically, FBXW11 is a substrate adaptor of the Skp1-cullin-F-box (SCF) ubiquitin ligase complex, which catalyzes phosphorylation-dependent ubiquitination [44]. Despite the important role of FBXW11 in human aging-related disease [45], little is known about the impact of FBXW11 on IDD. Here, LC‒MS/MS and co-IP revealed that FBXW11 negatively regulates TAK1 through K48-linked ubiquitination at the K446 residue. Thus, our findings indicated that miR-150-5p plays a pivotal role in modulating NPC senescence via the FBXW11/TAK1/NF-κB pathway. This provides fundamental insight into miR-150-5p as a promising target for attenuating IDD.

miRNA-based therapeutics are potential approaches for treating IDD [46]. Our prior studies revealed that miRNA-based therapy via the intradiscal injection of miRNA vectors into the intervertebral disc is a feasible and effective treatment strategy [10, 11]. However, miRNAs are sensitive to nuclease degradation, which makes it difficult for them to persist for a long period in target tissue. The polyanionic charge and large molecular weight are other obstacles for the application of miRNAs. [47] Thus, the choice of an appropriate delivery system is a prerequisite for miRNA-based therapies in vivo. In this study, we developed a novel therapeutic nanoparticle loaded with miR-150-5p and tested its targeting efficiency and tolerability both in vitro and in vivo. This approach has the following integrated advantages. First, by taking advantage of their small size and adaptable positive charge, our nanoparticles could prolong the half-life of miR-150-5p in vivo and maintain therapeutic amounts of miRNAs in intervertebral disc tissue. Second, the EY3 aptamer exhibits high affinity and specificity for NPCs, which could improve their intracellular uptake and therapeutic efficacy through receptor-mediated transport. Third, PLGA is a biocompatible polymer that is extensively used for drug delivery because of its long-term stability and sustained release efficacy. miR-150-5p encapsulated with PLGA can protect cells from nuclease degradation and decrease the immune response [48]. By taking advantage of its high drug loading and encapsulation efficiency, PLGA could protect encapsulated miR-150-5p from nuclease degradation and prevent it from being internalized by endo/lysosomes. Fourth, because of their anionic character, cellular membranes exhibit low permeability for miRNAs. Transfection efficiency and loading capacity could be enhanced by the binding of a positively charged PLL ligand to a negatively charged cellular membrane [49–51]. Therefore, based on the above advantages, PLGA-PLL-PEG nanoparticles may optimize the release mechanism of miR-150-5p. IDD often begins with cellular nutrient deprivation, progresses to forced anaerobic metabolism, and ultimately leads to a decreased pH within the intervertebral disc [52]. Under this acidic environment, PLL exhibits an enhanced protonation effect, and promotes the proton sponge effect, which significantly facilitates the successful endo/lysosomal escape of the miRNA-loaded nanoparticles. Once escaped from the endo/lysosomal compartments, the biodegradable properties of PLGA facilitate the intracellular release of miRNA, enabling it to participate in the gene regulation process. Importantly, our therapeutic nanoparticles, including PLGA dendrimers, PLL ligands, aptamers and miRNAs, could be readily produced by standard in vitro synthesis techniques. [53, 54] Furthermore, PLGA and PLL currently possess US Food and Drug Administration (FDA) approval for the delivery of a variety of drugs in humans [55, 56]. Therefore, our findings could lead to novel insights into the appropriate design of therapeutic nanoparticles with clinical efficacy against IDD in future investigations.

Various nanoparticles have been widely used for intradiscal injection for the treatment of IDD [31]. Intradiscal injection was found to act as a “double-edged sword” in treating IDD. Increasing clinical evidence showed the benefits of intradiscal injection on the disc repair and regeneration as they bypass the obstacles of the avascularity and the endplates [57–59]. Paradoxically, the use of needle puncture has been found to induce and accelerate disc degeneration [60, 61]. Some authors emphasized small diameter puncture needle and minimal dose of agent to avoid disc degeneration [62, 63]. Besides the trans-annular route, the trans-pedicular approach has recently been described as an alternative route for intradiscal injection, but the long-term effect on the endplate integrity has not yet been evaluated [64]. It suggests that the continuous development of biological technology may provide a new strategy of local drug delivery in IDD treatment.

One limitation of the present study should be taken into account. In this study, multiple intradiscal injections were used in the in vivo IDD rescue experiment. Considering the clinical translation, it might be optimal to adopt a single intradiscal injection with the sustained drug release and long-term efficacy. It could avoid the impairment of AF integrity and NP depressurization by multiple intradiscal injections. However, to date, most delivery strategies for a consecutive and controlled drug release are not compatible with single injection approaches [65]. Therefore, a single intradiscal injection is often not practical for IDD treatment. Further studies are needed to develop an injectable biomaterial that can both protect the therapeutic agents and allow for their sustained delivery, while avoiding repeated injections as these are detrimental to the AF and NP structure.

## Conclusions

In this study, we equipped the NPC-specific aptamer (EY3)-PLGA-PLL-PEG nanoparticles encapsulating miR-150-5p that could directly bind to the transmembrane protein FasL on the surface of NPCs, thereby accelerating the transport of nanoparticles to NPCs. From in vitro experiments to in vivo and preclinical studies, NPC-targeting nanoparticles delivering miR-150-5p show favorable therapeutic efficacy and safety and may constitute a promising treatment for IDD.

### Supplementary Information

Supplementary Material 1.

## Data Availability

The data that support the findings of this study are available from the corresponding author upon reasonable request.

## Abbreviations

AF: Annulus fibrosus
DMEM: Dulbecco’s modified Eagle’s medium
DLS: Dynamic light scattering
ECM: Extracellular matrix
FISH: Fluorescence in situ hybridization
IDD: Intervertebral disc degeneration
IHC: Immunohistochemistry
miRNA: microRNA
KO: Knockout
NP: Nucleus pulposus
NPC: Nucleus pulposus cell
PEG: Polyethylene glycol
PLGA: Poly(l-lactide-co-glycolide)
PLL: Poly-l-lysine
SASP: Senescence-associated secretory phenotype
SELEX: Systematic evolution of ligands by exponential enrichment
TEM: Transmission electron microscopy
WT: Wild-type
