# Supplementary material for: Effective delivery of miR-150-5p with nucleus pulposus cell-specific nanoparticles attenuates intervertebral disc degeneration
Source: J Nanobiotechnology. 2024 May 27;22:292. doi: 10.1186/s12951-024-02561-x (PMC11129471; doi:10.1186/s12951-024-02561-x)
Supplement: Supplementary file 1 — Supplementary Material 1. [file 12951_2024_2561_MOESM1_ESM.docx]

Supporting Information

**Effective delivery of miR-150-5p with** **nucleus pulposus cell-specific nanoparticles attenuates intervertebral disc degeneration**

Hua Jiang^1,2^*^✝^, Hongyu Qin^1^, Qinghua Yang^1^, Longao Huang^1^, Xiao Liang^1^, Congyang Wang^1^, Abu Moro^1^, Sheng Xu^3^, and Qingjun Wei^2^*^✝^

1 Department of Spine Surgery, The First Affiliated Hospital of Guangxi Medical University, Nanning 530021, Guangxi Zhuang Autonomous Region, P. R. China

2 Department of Orthopaedic Surgery, The First Affiliated Hospital of Guangxi Medical University, Nanning 530021, Guangxi Zhuang Autonomous Region, P. R. China

3 Research Centre for Regenerative Medicine, Guangxi Engineering Center in Biomedical Material for Tissue and Organ Regeneration, Guangxi Medical University

Nanning 530021, Guangxi Zhuang Autonomous Region, P. R. China


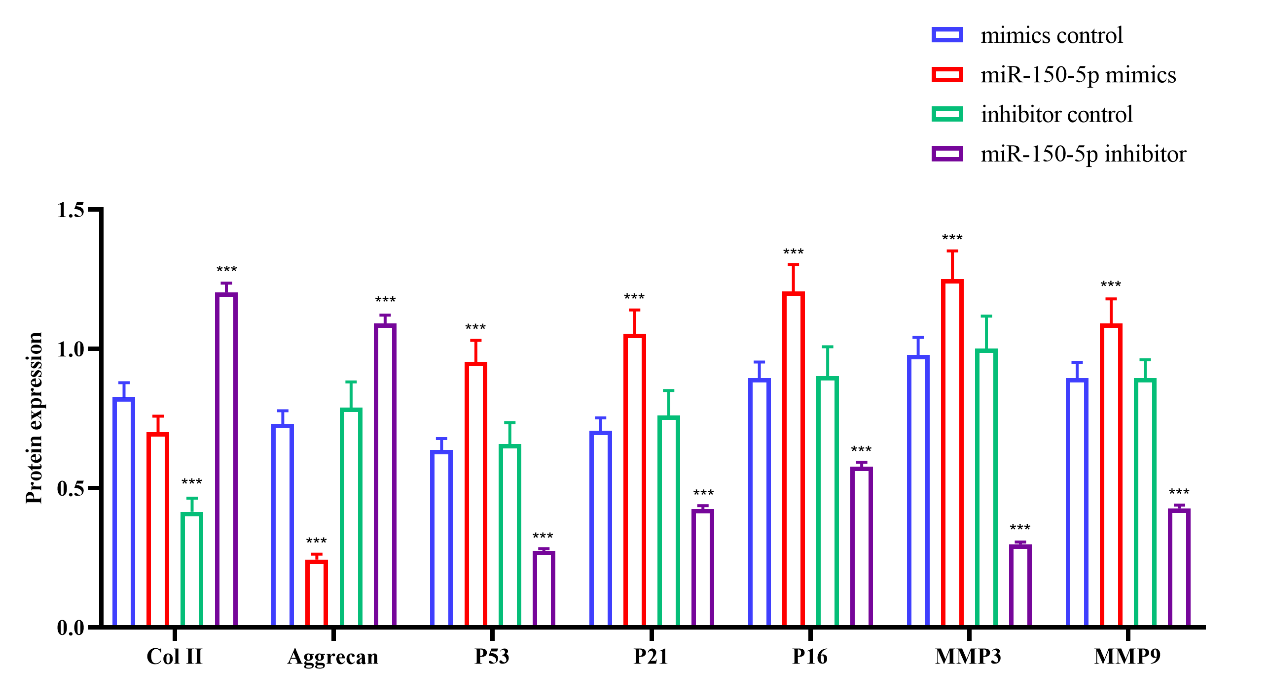


**Supplementary Figure 1.** Western blot analysis showed that the protein expression levels of P53, P21, P16, MMP3 and MMP9 were affected by the upregulation or downregulation of miR-150-5p.


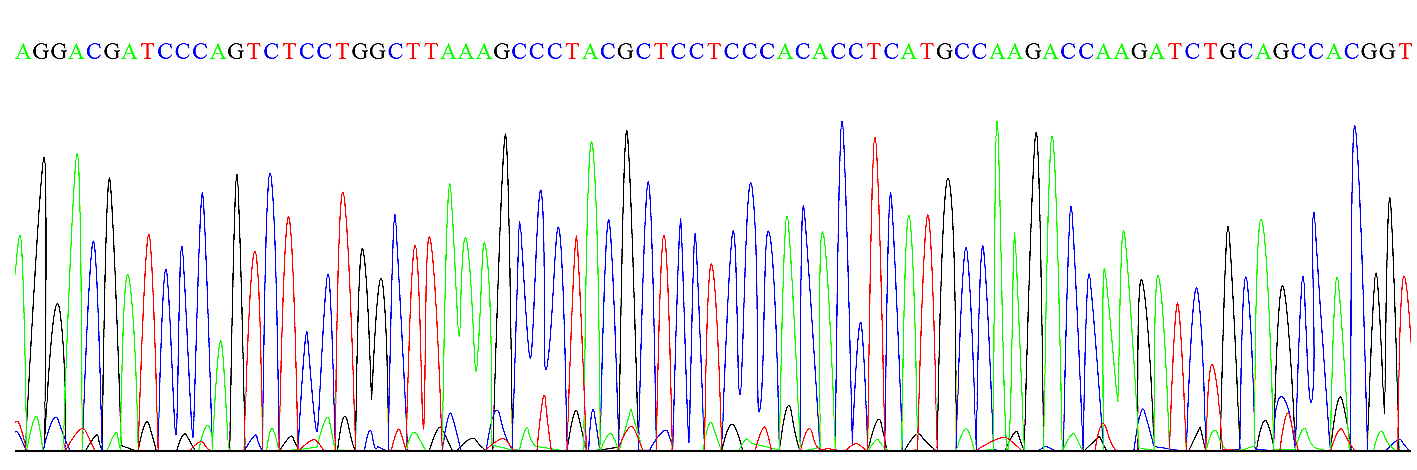


EY1


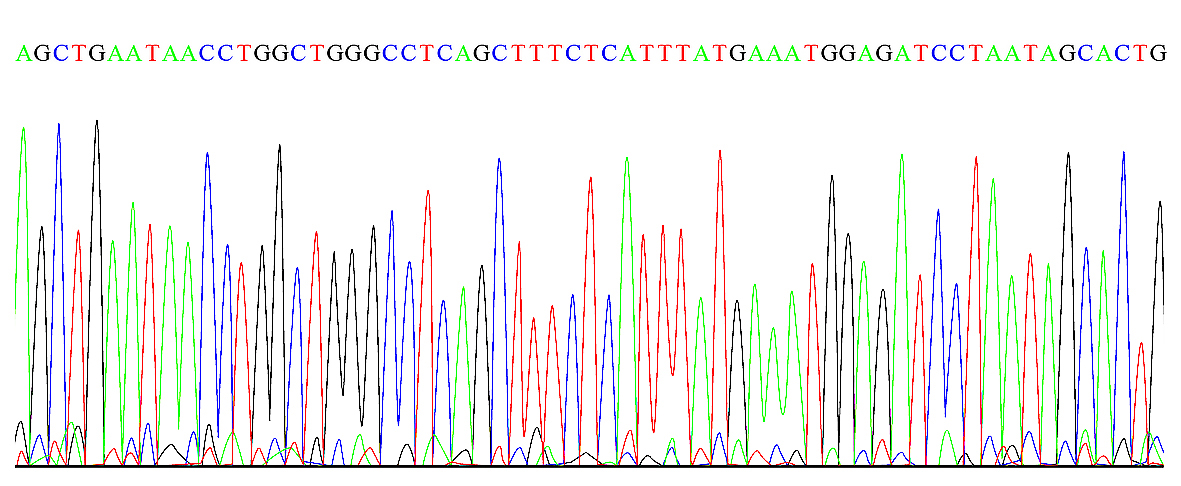


EY2


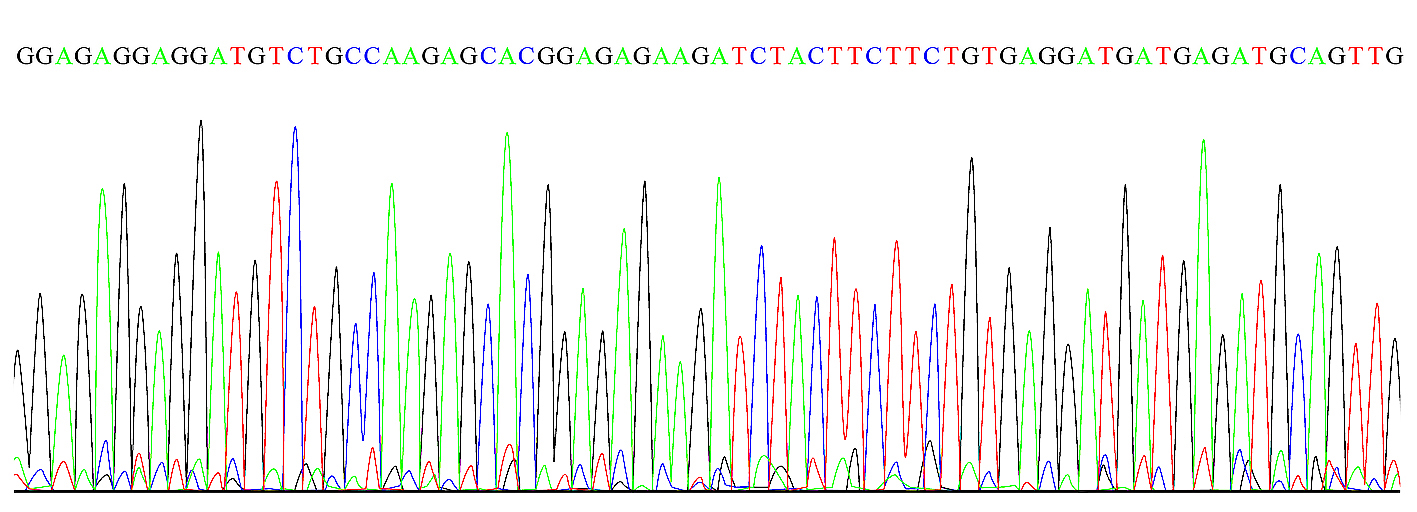


EY3


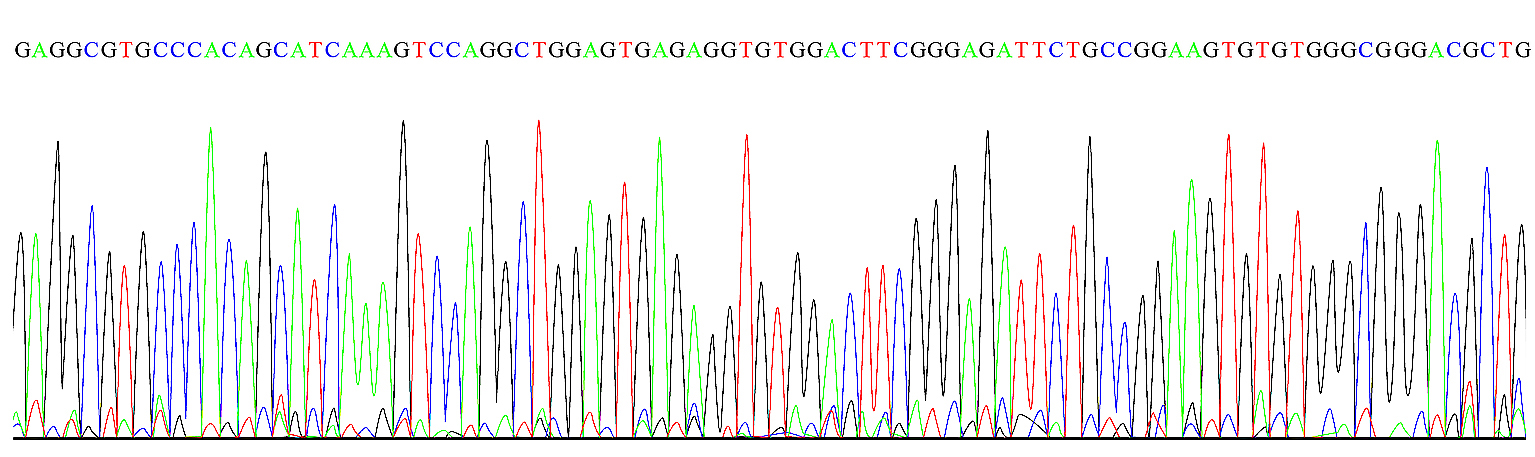


EY4

**Supplementary Figure 2.** The sequences of candidate aptamer EY1-4.


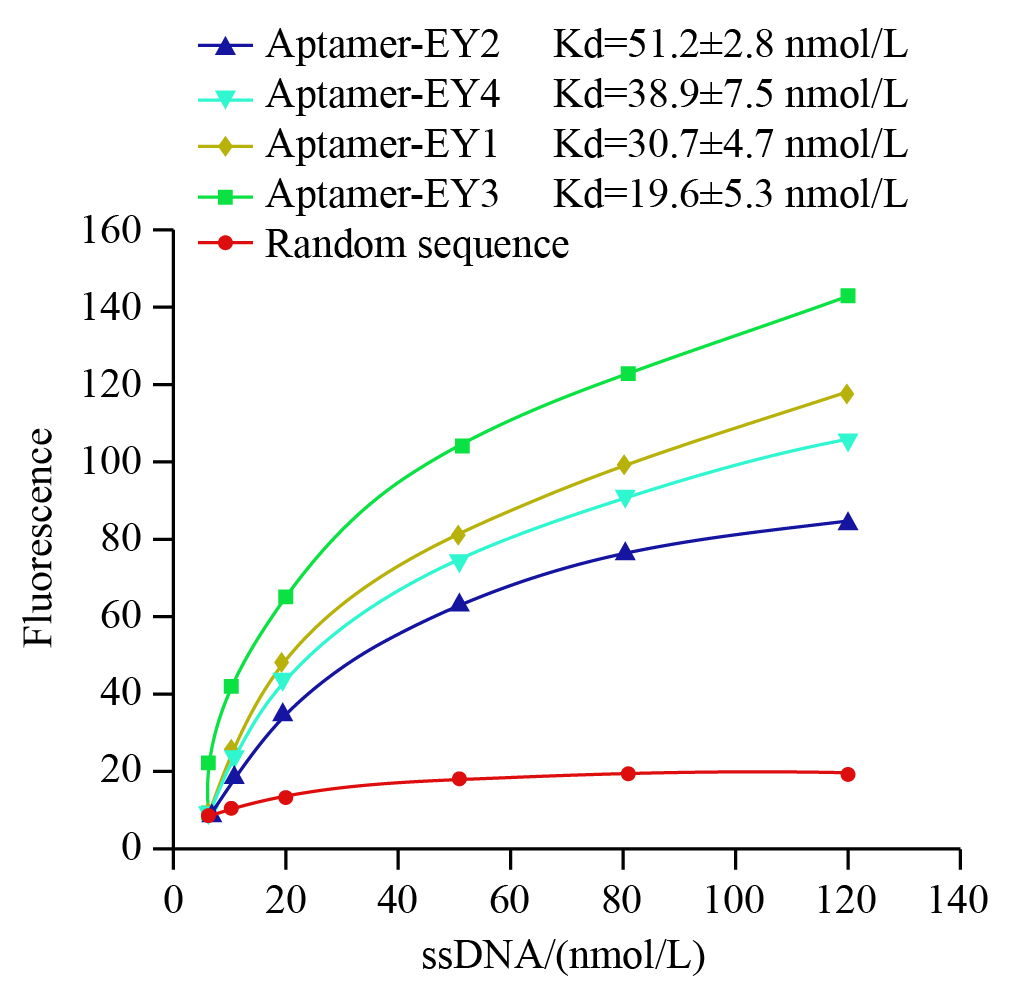


**Supplementary Figure 3.** Flow cytometry to determine the binding affinity of the aptamer candidates for nucleus pulposus cells.


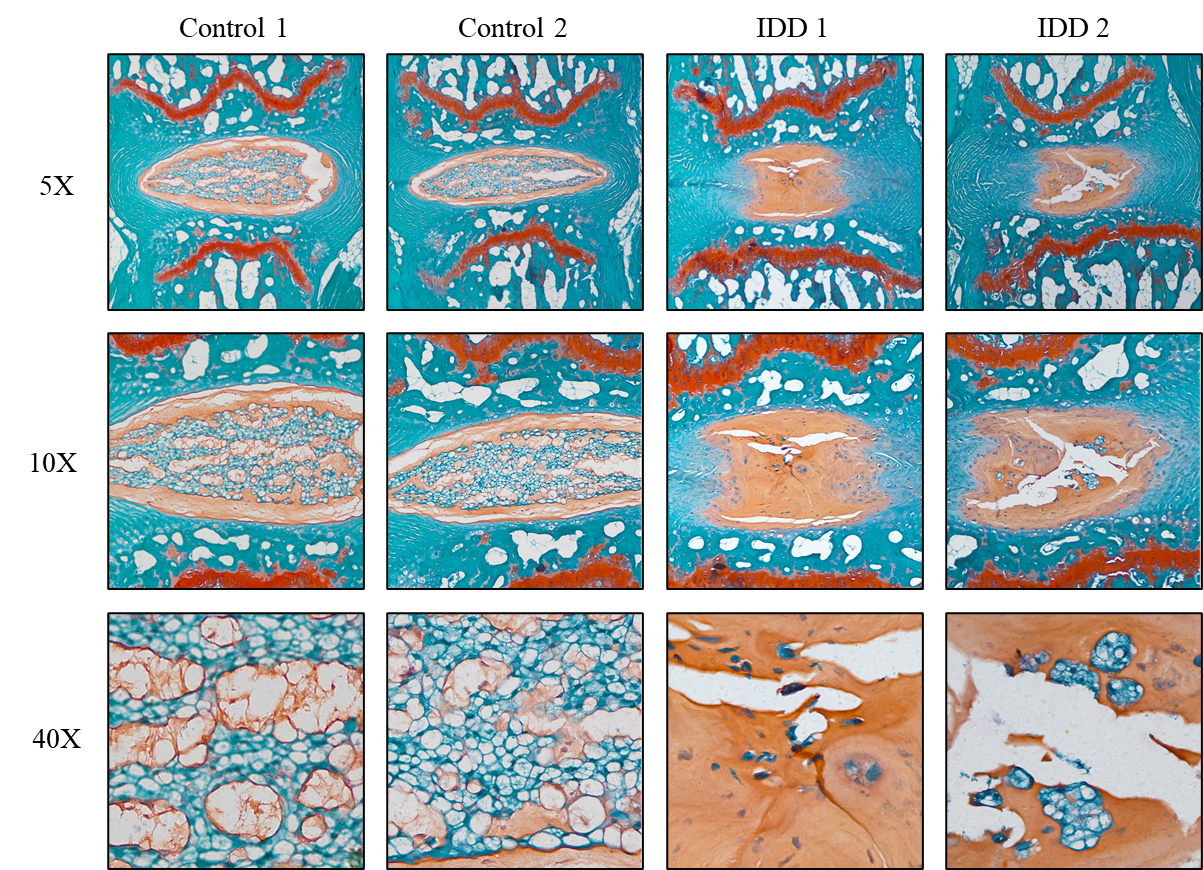


**Supplementary Figure 4.** Alcian Blue staining showing the significant intervertebral disc degeneration at 4 weeks after needle puncture surgery.

| **Supplementary Table 1. Characteristics of the study subjects from whom disc tissue used in qRT-PCR and histological examination** | | | |
| --- | --- | --- | --- |
| **Characteristics** | | **Healthy controls** | **IDD patients** |
| **Age (years)** | Median (range) | 54.2(24.8-60.0) | 53.9(23.6-66.3) |
| **Gender** | Male | 18 | 41 |
|  | Female | 12 | 26 |
| **Location of disc tissue** | L1/2 | 16 | 0 |
|  | L2/3 | 11 | 0 |
|  | L3/4 | 2 | 8 |
|  | L4/5 | 1 | 31 |
|  | L5/S1 | 0 | 28 |
| **Pfirrmann grade** | I | 28 | 0 |
|  | II | 2 | 0 |
|  | III | 0 | 20 |
|  | IV | 0 | 47 |
| qRT-PCR: real-time quantitative PCR; L: lumbar; S: sacral | | | |

| **Supplementary Table 2. Primer sequences used for qRT-PCR analysis** | | |
| --- | --- | --- |
| **Gene** | **Primer orientation** | **Primer sequence (5’-3’)** |
| **miR-150-5p** | Forward | ACACTCCAGCTGGGTCTCCCAACCCTTGTACCA |
| (Human) | Reverse | CTCAACTGGTGTCGTGGA |
| **miR-150-5p** | Forward | TCTCCCAACCCTTGTA |
| (Mouse) | Reverse | GAATACCTCGGACCCTGC |
| **FBXW11** | Forward | CGGGACTTTATCACTGCTTTA |
| (Mouse) | Reverse | ATCACTCGCTGCCATTCTTTA |
| **Col II** | Forward | GGGAATGTCCTCTGCGATGAC |
| (Mouse) | Reverse | CAGGCGCACCATCTCTGAT |
| **MMP13** | Forward | CTTCTTCTTGTTGAGCTGGACTC |
| (Mouse) | Reverse | CTGTGGAGGTCACTGTAGACT |
| **MMP3** | Forward | ACATGGAGACTTTGTCCCTTITG |
| (Mouse) | Reverse | TTGGCTGAGTGGTAGAGTCCC |
| **p21** | Forward | CCTGGTGATGTCCGACCTG |
| (Mouse) | Reverse | CCATGAGCGCATCGCAATC |
| **GAPDH** | Forward | GTCTCCTCTGACTTCAACAGCG |
|  | Reverse | ACCACCCTGTTGCTGTAGCCAA |

| **Supplementary Table 3. Differentially expressed miRNAs in NP tissues from IDD and controls in both one- and two-stage validation** | | | | |
| --- | --- | --- | --- | --- |
| **miRNAs** | **One-stage** | | **Two-stage** | |
|  | **Fold change** | **P-value** | **Fold change** | **P-value** |
| **Up-regulated** |  |  |  |  |
| mmu-miR-1970c | 2.7 | 0.12 | - | - |
| **mmu-miR****-1946b** | **5.1** | **0.08** | **-** | **-** |
| mmu-miR-7086-5p | 3.3 | 0.09 | - | - |
| mmu-miR-5133 | 2.8 | 0.16 | - | - |
| mmu-miR-7083-3p | 4.8 | 0.08 | - | - |
| mmu-miR-184-3p | 3.6 | 0.06 | - | - |
| mmu-miR-1900 | 4.2 | 0.12 | - | - |
| **mmu-****miR-125b-1** | **5.7** | **0.05** | **-** | **-** |
| mmu-miR-190a-5p | 4.2 | 0.09 | - | - |
| mmu-miR-34b | 3.9 | 0.37 | - | - |
| mmu-let-7a-5p | 3.2 | 0.19 | - | - |
| **mmu-miR-150-5p** | **7.8** | **0.001**** | **7.6** | **0.002**** |
| **mmu-****miR-6244** | **6.9** | **0.006**** | **5.3** | **0.16** |
|  |  |  |  |  |
| **Down-regulated** |  |  |  |  |
| mmu-miR-7076 | 0.13 | 0.15 | - | - |
| **mmu-****miR-1899** | **0.02** | **0.001**** | 0.05 | 0.10 |
| mmu-miR-5618-5p | 0.18 | 0.09 | - | - |
| mmu-miR-6396 | 0.23 | 0.27 | - | - |
| **mmu-****miR-21c** | **0.09** | **0.05** | **-** | **-** |
| mmu-miR-6237 | 0.11 | 0.31 | - | - |
| mmu-miR-7650 | 0.12 | 0.20 | - | - |
| mmu-miR-191-5p | 0.35 | 0.22 | - | - |
| mmu-miR-7081-3p | 0.10 | 0.12 | - | - |
| NP: nucleus pulposus; IDD: intervertebral disc degeneration; Mmu: mouse; ** P < 0.01 by  Mann-Whitney U test. | | | | |
